# Niche expansion and adaptive divergence in the global radiation of crows and ravens

**DOI:** 10.1038/s41467-022-29707-5

**Published:** 2022-04-21

**Authors:** Joan Garcia-Porta, Daniel Sol, Matt Pennell, Ferran Sayol, Antigoni Kaliontzopoulou, Carlos A. Botero

**Affiliations:** 1 Department of Biology, Washington University in St. Louis, St. Louis, MO USA; 2 CREAF, Centre for Ecological Research and Applied Forestries, Cerdanyola del Vallès, Catalonia 08193 Spain; 3 CSIC, Spanish National Research Council, CREAF-UAB, Cerdanyola del Vallès, Catalonia 08193 Spain; 4 Department of Zoology, University of British Columbia, Vancouver, BC Canada; 5 Centre for Biodiversity and Environment Research, Department of Genetics, Evolution and Environment, University College London, London, UK; 6 Department of Evolutionary Biology, Ecology and Environmental Sciences, and Biodiversity Research Institute (IRBio), Universitat de Barcelona, E-08028 Barcelona, Catalonia Spain

**Keywords:** Evolutionary ecology, Evolutionary theory

## Abstract

The processes that allow some lineages to diversify rapidly at a global scale remain poorly understood. Although earlier studies emphasized the importance of dispersal, global expansions expose populations to novel environments and may also require adaptation and diversification across new niches. In this study, we investigated the contributions of these processes to the global radiation of crows and ravens (genus *Corvus*). Combining a new phylogeny with comprehensive phenotypic and climatic data, we show that *Corvus* experienced a massive expansion of the climatic niche that was coupled with a substantial increase in the rates of species and phenotypic diversification. The initiation of these processes coincided with the evolution of traits that promoted dispersal and niche expansion. Our findings suggest that rapid global radiations may be better understood as processes in which high dispersal abilities synergise with traits that, like cognition, facilitate persistence in new environments.

## Abstract (web summary)

Traits that facilitate adaptive responses to novel environments may facilitate global radiations. Here, the authors describe diversification dynamics of crows, finding that their global radiation coincides with high rates of phenotypic and climatic niche evolution.

## Introduction

**Fig. 1:**
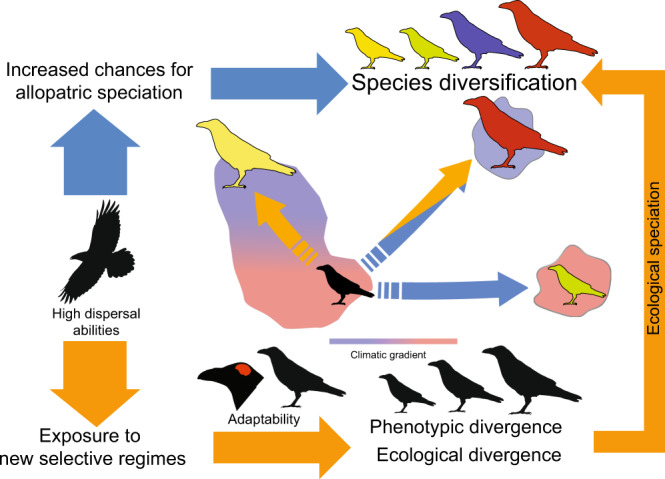
Conceptual depictions of the contributions of dispersal abilities and adaptability in (global) diversifications. Increased capacity to disperse over long distances creates more frequent opportunities for allopatric speciation (blue arrows) and exposes lineages to new environments (orange arrows). Traits that facilitate survival and local adaptation under suboptimal conditions prevent local extinction upon arrival and give lineages the opportunity to evolve in response to optimising selection from the new environmental conditions. These adaptive processes further increase the chances of diversification through ecological speciation.

Rapid global radiations –defined as rapid evolutionary diversifications of lineages that are accompanied by the colonisation of a large fraction of the planet– have been documented in nearly all domains of Life^1–3^. Despite representing much of current diversity, it is presently unclear why some clades only radiate regionally whereas others become global in similarly short timescales. Current understanding suggests that global radiations are facilitated by exceptional dispersal abilities^4–7^. These allow the expansion of species ranges and increase the frequency of long-distance dispersal events (e.g. to remote islands or between continents), multiplying the opportunities for allopatric speciation (Fig. 1). However, global radiations often occur across highly dissimilar environments (e.g. across large climatic gradients^7^) and may therefore involve exposure to resources, competitive regimes, predation pressures and climatic conditions that can largely differ from those experienced in the past. Thus, we expect that rapid global radiations may not only be facilitated by a capacity to disperse over long distances, but also by an exceptional ability to respond to new environmental challenges^8,9^. If so, global radiations should involve a considerable amount of adaptive diversification driven by environmental differences (for example, through ecological speciation^10–12^) (Fig. 1).

Here, we investigate the importance of adaptive processes in global radiations, asking whether they are associated with niche expansions and parallel pulses of rapid speciation and phenotypic divergence^10,13^. We address these questions in the genus *Corvus* (crows and ravens), a prominent group within the avian family *Corvidae* that has expanded across the world occupying almost all of Earth’s biomes (from hottest deserts to the arctic regions), branching out into at least 46 distinct species^14^. In sharp contrast, the diversification of all other genera in *Corvidae* has resulted in far fewer species (≤20 species) and the colonisation of geographically restricted regions of the planet^15^. We begin by building a time-calibrated phylogeny of the superfamily *Corvoidea* to assess whether the diversification of *Corvus* is a fast global radiation. Having shown that the genus exhibits significantly faster speciation rates than its background clade, we then ask whether the rapid accumulation of new species in *Corvus* was coupled with a burst of morphological and ecological diversification. To further interpret the results, we also investigate whether the predicted burst of diversification was preceded by the acquisition of traits that facilitate not only dispersal but also the invasion of new ecological niches. Specifically, we focus on body size and encephalization, traits that in birds are thought to increase competitive ability^16^, tolerance of novel conditions^17,18^ and improve the capacity to exploit novel ecological opportunities^19,20^. Our findings across a variety of ecological and phenotypic traits are consistent with the notion that the global radiation of crows and ravens was enabled by the joint evolution of an exceptional capacity for long-distance dispersal and an equally impressive ability to exploit new ecological opportunities and adapt to them.

## Results

### Molecular phylogeny

Our time-calibrated phylogeny of the superfamily Corvoidea (Supplementary Fig. 1, see Methods), the parent clade that contains Corvidae, recovered well-supported relationships between species and was highly consistent with previously proposed phylogenetic hypotheses^21^. Our dating estimates indicate that Corvidae started to diversify between 18 and 22 Ma, and that *Corvus* began radiating between 8.8 to 11.3 Ma (around 10 Ma). These findings confirm earlier estimates, which place the *Corvus* radiation on a similarly short timescale^21–23^ and are consistent with the earliest fossils that can unambiguously be assigned to the genus *Corvus*^24,25^.

### Dynamics of species diversification

**Fig. 2:**
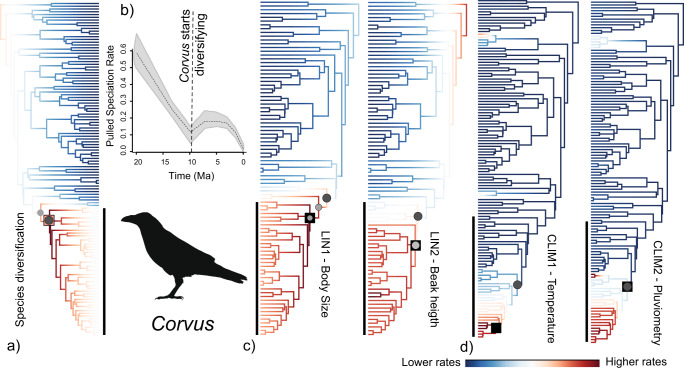
Rates of species, phenotypic and climatic diversification in Corvidae. Family-wide variation in rates of **a** species diversification, **c** phenotypic diversification, and **d** climatic diversification as estimated with BAMM. Circles indicate the alternative position of shifts in shift configurations with a frequency higher than 0.2 for linear measurements and 0.05 for climate (diameter and grey shading is proportional to the frequency). Squares indicate shifts detected by MEDUSA (red) and MOTMOT (black). LIN1 = body size; LIN2 = beak height; CLIM1 = temperature gradient; and CLIM2 = precipitation gradient. **b** Speciation rates through time as estimated by pulled speciation rates. Grey shaded area depicts 95% credible intervals.

We applied BAMM^26^ and MEDUSA^27^ on the maximum clade credibility tree (hereafter MCC tree) to ascertain whether *Corvus* exhibited different diversification dynamics than the rest of Corvidae. In BAMM, we found that the best-supported shift configurations involved a single rate increase either at the base of *Corvus* (frequency = 0.36) or at the node separating *Corvus* from its sister genus *Coloeus* (frequency = 0.28) (Fig. 2a and Supplementary Fig. 2a). Faster rates of diversification in *Corvus* were also detected when comparing mean rates across the posterior set of trees (Supplementary Fig. 3a). Similarly, a cohort analysis^28^ confirmed that *Corvus* exhibits macroevolutionary dynamics well detached from all other Corvidae (Supplementary Fig. 4a). Consistent with these findings, BAMM analyses indicate a significant decay in speciation rates within Corvidae, conspicuously interrupted by a secondary peak of speciation around 10 Ma, the estimated time of origin of *Corvus* (Supplementary Fig. 2c). MEDUSA analyses on the MCC tree and on the posterior set of trees also indicate that *Corvus* is at least double the mean background diversification rate of its family (Supplementary Figs. 2b, 5a).

BAMM and MEDUSA can robustly identify nodes or areas within a tree where diversification rates have shifted but are unable to distinguish the exact combination of speciation and extinction that may have driven such shifts^29^. Thus, we confirmed the existence of heterogeneity in diversification dynamics within Corvidae by evaluating changes in the dynamics of pulled speciation rates^29^ (hereafter PSR). We note that PSR variation over time is completely identifiable, given that PSR do not depend on parametric assumptions about speciation and extinction rates and all models in a congruent set—as defined by^29^—share a common PSR curve. As such, analysis of PSR variation is a robust complementary approach to BAMM and MEDUSA analyses. Visualisation of PSR variation through time within the family Corvidae confirms our initial findings (Supplementary Fig. 2c). Specifically, we observe a decay in speciation rates over time interrupted by a secondary peak of speciation around 10 Ma, the estimated time of origin of *Corvus* (Fig. 2b). Thus, our results suggest that, at a minimum, *Corvus* exhibited significantly different diversification dynamics than its background clade and was able to accumulate a much higher number of species per unit of time than closely related genera.

### Dynamics of phenotypic diversification

If the observed burst of species diversification in *Corvus* resulted from adaptive processes (e.g. ecological speciation), then we predict that this burst should be associated with a parallel increase in eco-morphological diversification^10,13^. We begin testing this prediction through an analysis of various morphological traits with well-proven locomotory and ecological relevance^30–35^. Our list of traits includes linear measurements of two elements of the upper limbs (i.e. humerus and ulna), three elements of the lower limbs (i.e. femur, tibiotarsus and tarsometatarsus) and the length, width, and height at the middle point of the upper beak (Supplementary Data file 1). We also characterised the shape variation of the beak through geometric morphometrics (Supplementary Data file 2). Linear and geometric morphometrics datasets were analysed independently by generating separate morphospaces through phylogenetic principal components analyses, *p*PCA^36^. Both of these *p*PCAs were individually optimised under the models of evolution that presented the best relative fit^37^.

**Fig. 3:**
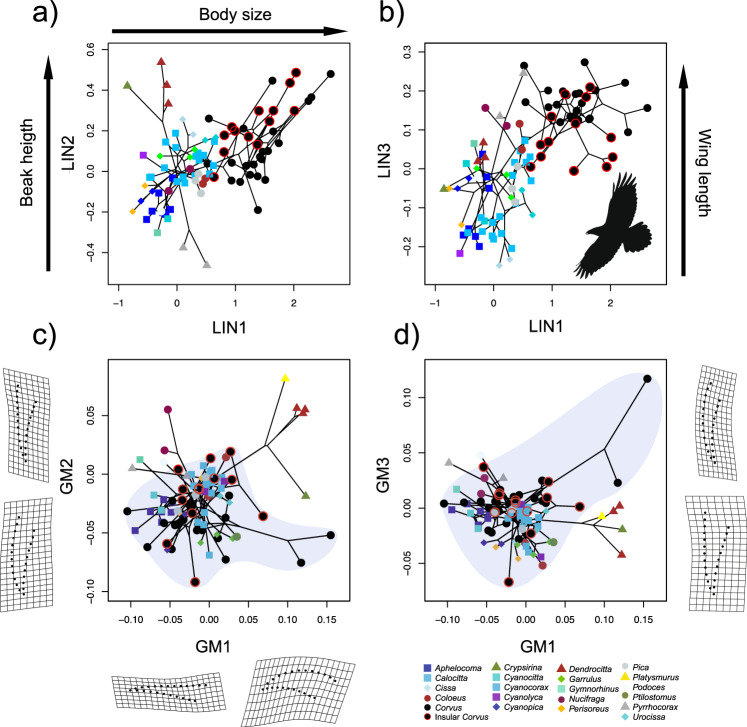
Morphological diversity in the family Corvidae. Upper panels **a**, **b** depict the morphospace derived from a phylogenetic PCA on linear measurements. Lower panels **c**, **d** depict the morphospace of beak shape derived from a phylogenetic PCA on data obtained through geometric morphometrics. Black lines depict phylogenetic relationships among species. Blue regions highlight the extent of the beak morphospace occupied by *Corvus*. Peripheral graphs in the bottom panels depict thin-plate spline deformation grids to help visualise extreme shapes along each axis.

The *p*PCA of linear measurements was optimised with a BM model of evolution (Supplementary Table 1). The first three components in this analysis (i.e. LIN1–LIN3) collectively explained 94% of the variation in linear measurements and reflected functionally relevant variation in body size, beak dimensions (mainly height), and relative wing length (Supplementary Table 2). Within these components, the genus *Corvus* exhibits the greatest level of morphological variation among Corvidae, particularly in LIN1 and LIN2. Body size (LIN1) is on average larger in *Corvus* than in other Corvidae and shows twice as much variation as the second most variable genus of the family (i.e. *Cyanocorax*) (Fig. 3a). As for variation in beak height (LIN2), *Corvus* also exhibits the greatest disparity in its family, being 2.75 times greater than what is observed in the second most variable genus (i.e. *Cyanocorax*) (Fig. 3a). In birds, variation in both traits (body size and beak shape) is linked to differences in ecologies, including resource partitioning^9,38,39^ and shifts in climatic niche^40–42^ and therefore, the increase in phenotypic disparity observed in *Corvus*, likely reflects an increase in ecological diversification. Critically, we find evidence that wing shape—measured as 'hand-wing index' (HWI), a well-known proxy for wing aspect ratio and dispersal ability in birds (see^43^ and references therein)— significantly covaries with both body size (LIN1) and relative wing length (LIN3) (*F* = 4.21, *p* value <0.001 and *F* = 40.56, *p* value <0.001, respectively, Supplementary Fig. 6). This covariation confirms that *Corvus* has not only longer wings but also a greater potential for long-distance dispersal than most of its close relatives.

We used three complementary approaches to investigate whether the observed morphospace expansion in *Corvus* was indeed accompanied by accelerated rates of phenotypic evolution: BAMM^44^, MOTMOT^45^ and BROWNIE^46^. Each of these methods relies on different analytical approaches to test for rate heterogeneity across a tree. BAMM and MOTMOT are designed to identify the most likely position and magnitude of rate shifts in a phylogeny with no a-priori assumptions regarding shift locations. BROWNIE, on the other hand, enables testing alternative hypotheses on potential rate shifts at specific nodes of the tree (e.g. the split between *Corvus* and all other Corvidae). Our results are consistent across all three alternatives. In our analysis of body size (LIN1), both BAMM and MOTMOT detected a single rate increase, either at the base of *Corvus* (MOTMOT and BAMM shift configuration frequency = 0.2), at the split between *Coloeus* and *Corvus* (BAMM shift configuration frequency = 0.23), or at the split between *Nucifraga* and the *Coloeus*/*Corvus* clade (BAMM shift configuration frequency = 0.28) (Fig. 2c and Supplementary Fig. 7). These findings, as well as a related cohort analysis (Supplementary Fig. 4b), suggest that acceleration of body size evolution was initiated either at or slightly before the origin of *Corvus*. We note, however, that regardless of the actual location of that shift, mean rates of body size evolution were higher in *Corvus* than in the neighbouring clades in both the MCC tree and in the posterior set of trees (Fig. 2c and Supplementary Figs. 3b, 5b, 7). Accordingly, we used the BROWNIE approach to compare a single rate model against a two-rates model with a shift at the node that separates *Corvus* from all other Corvidae. Likelihood-ratio tests indicate that the two-rates model is always better supported across the entire posterior set of trees. When we compute rates according to the two-rates model, these are significantly higher in *Corvus* than in other Corvidae (Supplementary Fig. 8a).

In our analyses of beak height (LIN2), both BAMM and MOTMOT detected a single sharp rate increase either at the base of *Corvus* or within it, specifically, in the clade that contains most of the species in the genus (Fig. 2c and Supplementary Figs. 4c, 7). Once again, this finding was confirmed by the observation that rates of LIN2 evolution are higher in *Corvus*, both within the MCC tree and within the posterior set of trees (Supplementary Figs. 3b, 5b, 7). A similar pattern was also observed with BROWNIE (Supplementary Fig. 8b). In sharp contrast, neither BAMM, MOTMOT nor BROWNIE detected rate heterogeneity in the evolution of relative wing length, LIN3, either within the MCC tree (Supplementary Figs. 4d, 7), or across the posterior set of trees (Supplementary Figs. 3b, 5b, 8c).

We then explored the evolution of the beak shape using a geometric morphometric approach. A *p*PCA of superimposed landmark coordinates (see methods) optimised with an Early Burst model of evolution (Supplementary Table 1), indicates that *Corvus*-specific variation in beak shape either resembles (area of the α-convex hull in GM1 - GM2 morphospace: *Corvus* = 0.017; other Corvidae = 0.024; Fig. 3c) or even surpasses that of all other Corvidae (area of the α-convex hull in GM2 - GM3 morphospace: *Corvus* = 0.019; other Corvidae = 0.014; Fig. 3d). In agreement with previous analyses using linear beak measurements, BAMM and MOTMOT indicate that the diversification of beak shape also accelerated within *Corvus* (Supplementary Figs. 9, 10). Such rate increases were consistently observed with all other methods and across our entire posterior set of trees (Supplementary Figs. 3c, 5c, 11). Follow up analyses with a phylogenetic MANOVA of centroid sizes on superimposed landmark coordinates indicated that beak size and beak shape components covary (Pillai´s trace = 0.64, *p* value = 0.01). To address this issue, we recomputed the *p*PCA and the rates of evolution on allometric-free beak shape residuals. Although lower in magnitude, these new metrics produced qualitatively identical patterns to those obtained with the initial shape components (Supplementary Figs. 3d, 5d, 12–15). Based on these findings, we conclude that even though some of the evolution of beak shape in *Corvus* was likely a reflection of changes in beak size (and likely, body size), there is nevertheless strong evidence indicating that *Corvus* beaks also responded independently to selection during the radiation process.

**Fig. 4:**
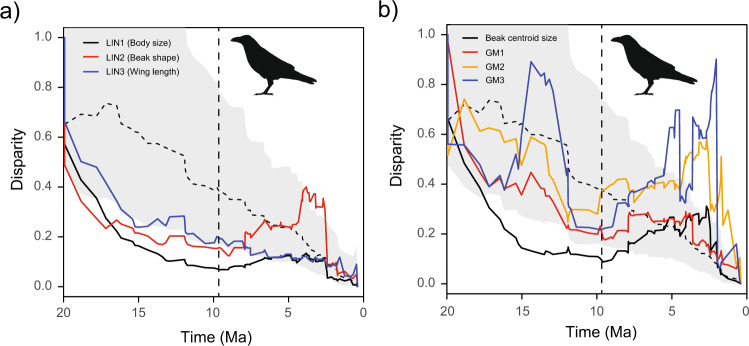
Evolution of phenotypic disparity in Corvidae. Mean subclade disparity through time (DTT) for **a** linear measurements and **b** beak shape obtained through geometric morphometrics. Dashed lines indicate median subclade DTT based on 1000 simulations of character evolution under a Brownian motion model with a single tree-wide rate of change. Grey shaded areas depict 95% credible intervals for DTT estimated in simulation. Vertical dashed lines indicate the onset of diversification of the genus *Corvus*.

We further assessed temporal dynamics of morphological diversification in Corvidae by plotting phenotypic disparity through time (DTT)^47^. Specifically, we plotted the mean subclade disparity at each node of the phylogeny against node age and compared it against a null model with a pure Brownian Motion (BM) model of evolution and a single rate for the entire tree. This null model specifically estimates the amount of disparity that can be expected from a simple process of stochastic evolution^48^ and therefore serves as an appropriate baseline for comparison. Empirical DTT plots indicate that most morphological traits exhibited a clear decrease in phenotypic disparity over the first half of the diversification of the family Corvidae (Fig. 4a, b). However, most of these decays were conspicuously interrupted by new pulses of phenotypic disparity that began ca. 10 Ma (i.e. at the origin of *Corvus*). These empirical patterns are clearly different from the Brownian expectations based on a single model of evolution, which shows a linear decrease through time (Fig. 4a, b for findings on the MCC tree and Supplementary Fig. 16a, b for confirmation across the posterior set of trees).

To confirm that these effects were indeed produced by the acceleration of rates of evolution in *Corvus*, we simulated phenotypic disparity using the rate variation estimated by BAMM for LIN1, LIN2, GM1, GM2 and GM3 (i.e. the traits for which BAMM detected *Corvus*-specific rate accelerations). Reassuringly, the medians and 95% confidence intervals (CI) of these simulations are broadly consistent with the observed patterns of divergence in all traits (Supplementary Fig. 17a, b). Additionally, we note that when empirical DTT plots were recomputed excluding *Corvus* from the phylogeny, we did not observe any secondary pulses in disparity (Supplementary Fig. 18a, b). These two findings strongly indicate that the increase in phenotypic disparity observed in Corvidae ca. 10 Ma can be specifically attributed to the acceleration of phenotypic evolution within *Corvus*.

### Geographic variation and dynamics of climatic diversification

**Fig. 5:**
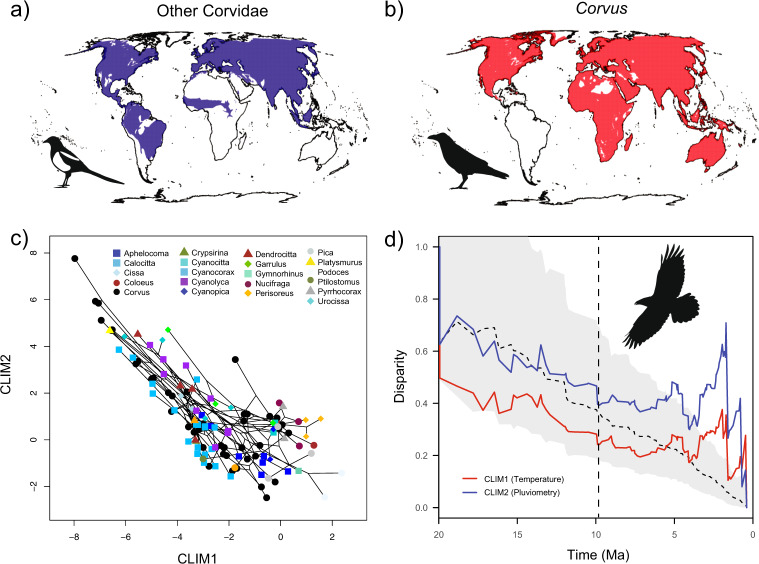
Distributions and climatic niche of Corvus and Corvidae. World distributions of **a** all species of Corvidae excluding *Corvus* and **b** all species of *Corvus*. **c** Climatic space occupied the family Corvidae. Black lines depict phylogenetic relationships among species. **d** DTT for climatic diversification in the family Corvidae. The vertical dashed line indicates the onset of diversification of the genus *Corvus*. Grey shaded areas depict 95% credible intervals for DTT estimated in simulation.

Our analyses indicate that the genus *Corvus* recolonised most of the areas occupied by other Corvidae and collectively extended the geographic distribution of the family by almost 30% as it moved into regions like Australasia, northern and southern Africa, Madagascar and the Arabian Peninsula (Fig. 5a, b). This massive geographic expansion is even more remarkable when considering that the second most widely distributed genus in Corvidae (i.e. *Pica*) occupies only a third of the area occupied by *Corvus* (Supplementary Fig. 19).

We took advantage of the vast amount of georeferenced records for Corvidae in eBird^49^ to investigate whether the geographic expansion of *Corvus* was accompanied by a parallel expansion in the climatic niche. We used a principal components analysis^50^ to describe the main axes of variation in the climate space occupied by all Corvidae. Of the two main axes identified, the first one, CLIM1, captured primarily a temperature gradient, whereas the second one, CLIM2, captured a gradient in precipitation (Supplementary Table 3). Visualisation of scores of these two axes revealed that while most of the genera in Corvidae occupy relatively small climatic niches, *Corvus* is broadly distributed in climate space (Fig. 5c). Even more striking, the climatic niche of *Corvus* almost completely encompasses that of other Corvidae and further expands it toward warmer (i.e. more negative values in CLIM1, Fig. 5c), wetter (i.e. more positive values in CLIM2, Fig. 5c) and drier habitats (i.e. more negative values in CLIM2, Fig. 5c). Not surprisingly, the rates of evolution estimated for both climate axes are generally elevated in *Corvus* as compared to the rest of the family (Fig. 2d and Supplementary 20–22). Just as with morphology, the DTT plots for climatic niche components indicate that increasing levels of climatic disparity were primarily produced during the last 10 Ma (Fig. 5d). Here too, these patterns are robust to phylogenetic uncertainty (Supplementary Figs. 3e, 5e, 16c), and better approximated by a model in which rates of evolution are assumed to be accelerated in *Corvus* (Supplementary Fig. 17c). In this case, though, significant levels of disparity are still detected in recent times if *Corvus* is excluded from the analysis (Supplementary Fig. 18c), highlighting the fact that a remarkable amount of climatic niche evolution is also observed in the genera *Pica* and *Urocissa* (see Supplementary Fig. 20). On a related note, we also found that the genus *Corvus* exhibits the highest number of sympatric species among Corvidae, which indicates that it could have also experienced higher levels of interspecific competition than its closest relatives (Supplementary Fig. 23).

### Phenotypic drivers of global radiation

**Fig. 6:**
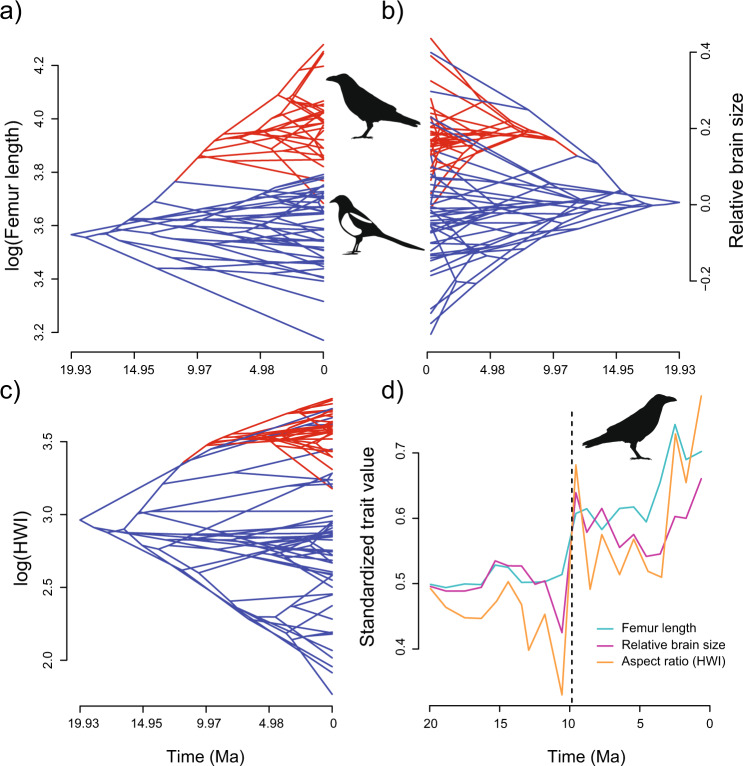
Evolution of body size (as estimated from femur length), wing shape (as estimated by “hand-wing index”, HWI), and relative brain size in the family Corvidae. The vertical positions of nodes in phenograms **a**–**c** depict the reconstructed values of a given trait, whereas their horizontal position (i.e., time of divergence) and connectivity reflect the underlying phylogeny (*Corvus* branches are highlighted in red). **d** Clade-wide patterns of variation in the mean ancestral body size, HWI, and relative brain size through time.

To assess whether the remarkable ecological and lineage diversification of *Corvus* was potentially triggered, as predicted, by the joint evolution of exceptional capacities to both disperse and respond to ecological challenges, we now take a closer look at the traits that help distinguish *Corvus* from other Corvidae. We previously showed that *Corvus* differs from most of its close relatives in two key traits related to long-distance dispersal and competitive ability: large bodies and long wings relative to the body (Fig. 3a, b). Additionally, extensive natural history accounts and ethological experiments suggest that *Corvus* may also excel in cognitive ability (e.g. refs. ^51,52^), a feature that is known to increase the ability to tolerate novel environmental conditions^17,18^ and exploit novel ecological opportunities^19^. Relative brain size provides a principled way to compare cognitive capacity across bird species, as it is known to correlate positively with behavioural flexibility^53^, learning^54^, memory^55^, neuron number^56^, and the volume of pallial areas associated with general-domain cognition^57^. We thus evaluated whether body size, relative wing length, and relative brain size increased in value with the origin of *Corvus* (around 10 Ma) and maintained comparatively higher values during the early burst of diversification of this genus. Both predictions are strongly supported by our data. Specifically, morphological comparisons show that crows and ravens exhibit larger relative brain sizes than other Corvidae (phylogenetic ANOVA with Pagel’s lambda = 0.75, *F* = −0.15, *p* value = 0.054; Supplementary Fig. 24), as well as larger bodies and higher HWI (see results in previous sections). Moreover, ancestral state reconstructions detect a substantial increase in mean relative brain size, HWI, and body size at the origin of *Corvus* and indicate that these traits maintained comparatively higher values within this genus throughout its entire radiation process (Fig. 6a–c). Importantly, the initial increase in reconstructed values and the maintenance of such high values thereafter are also visible in family-wide averages (Fig. 6d) when *Corvus* is included in these metrics (compare with Supplementary Fig. 25 for when it is not). Additionally, we note that neither BAMM, MOTMOT nor BROWNIE detect significant rate shifts in relative brain size within Corvidae (Supplementary Fig. 26). Although diversification in body size significantly increased in *Corvus*, most of the ancestral and current body sizes in *Corvus* are bigger than all other Corvidae (Figs. 2a, 6a).

## Discussion

Our analyses show that in ~10 Ma, crows and ravens experienced a massive geographic expansion that allowed them to reoccupy and even surpass the already broad climatic niche of other Corvidae. In parallel with this geographic and niche expansion, *Corvus* experienced accelerated rates of species diversification accompanied by a remarkable fast expansion of the morphospace. *Corvus* for instance exhibits the greatest amount of body size variation and the highest rates of body size evolution in Corvidae. In terms of beak shape, crows and ravens not only reproduced many of the shapes already present in their family but also evolved entirely new beak types in short time periods (e.g., *Corvus crassirostris* and *Corvus moneduloides*). Because these morphological traits have a well-established adaptive basis^9,38,39^, our findings suggest that the global radiation of crows and ravens cannot merely be understood as the result of dispersal and (non-adaptive) allopatric speciation, but also of considerable adaptive divergence driven by ecological factors^10–12^.

The exact ecological and geographic processes behind the *Corvus* diversification are yet to be determined. One possibility is that the colonisation of vastly different climates induced adaptive diversification in body size (e.g., “Bergmann’s rule”^40^) and beak morphology^41,42^. Additionally, it is possible that some of the morphological divergence observed in *Corvus* was driven by the encounter with new ecological opportunities on islands^58,59^. Consistently with this interpretation, *Corvus* exhibits the highest rate of island colonisation in its family (including at least 15 insular endemics) and is one of the few corvids that reached remote archipelagos like Hawaii (3800 km from the mainland), Guam (1800 km from the mainland) and New Zealand (1700 km from the mainland). Additionally, insular species tend to occupy the periphery of *Corvus*’ morphospace and have produced some of its most divergent beak shapes (Fig. 3a, c, d). Given that many *Corvus* are currently sympatric (Supplementary Fig. 23), some fraction of the observed divergence could also reflect character displacement driven by interspecific competition^60^.

The remarkable key adaptations behind the outstanding dispersal ability of *Corvus* and its capacity to tolerate new environmental conditions and invade new ecological niches are also insufficiently understood. Our results suggest a number of potential candidates: elongated wings, bigger bodies, and larger relative brains. Elongated wings are generally correlated with an enhanced potential for dispersal among birds^61^ and Corvidae in particular (Supplementary Fig. 6). Large bodies confer significant advantages in interspecific competition^16^, and may therefore represent a significant asset during range expansion^16^. A large brain, relative to body size, provides the neural basis for behavioural innovation and learning, features that are known to facilitate persistence in new environments and the adoption of new resource opportunities^17,62^. We note that the rapid radiation of *Corvus* was preceded by the evolution of all three of these traits (Fig. 6), and therefore conclude that it is plausible that they may have triggered the rapid diversification of the clade.

The exceptional behavioural flexibility of *Corvus* is particularly evident in its extant species, which collectively display the greatest number of behavioural innovations reported for any avian genus^63^ and are frequent colonisers of human cities^64^ thanks in part to their ability to exploit new resources^65^ and their flexibility in nest site choices^66^. This exceptional behavioural flexibility could nevertheless challenge the idea that exposure to divergent selection played a major role in the diversification of the clade. Specifically, behavioural flexibility (just as phenotypic plasticity^67^) is often portrayed as a potential inhibitor of evolutionary change because it increases an individual’s ability to survive and reproduce even with an inappropriate phenotype^68–71^. How, then, could the early evolution of large-relative brain sizes have subsequently facilitated the evolution of new phenotypes in *Corvus*? The answer to this question could lie in theoretical and empirical work that suggests that although behavioural flexibility can reduce the strength of selection, it cannot avoid it altogether, especially when the new selective pressures are very different from those that a species faced in its ancestral niche^12,69,72–80^. Thus, it is possible that exceptional capacities for behavioural flexibility enabled ancestral *Corvus* to colonise habitats that were very different than the ones they had most recently evolved in, and that their ability to persist under suboptimal conditions allowed selection to subsequently improve the match between their phenotypes and the new environment^81,82^.

In conclusion, we have shown that the global radiation of crows and ravens was characterised by bursts of phenotypic and species diversification associated with parallel expansions of geographic ranges and ecological niches. ﻿Because colonisation success is often limited by ecological factors^12^, these findings suggest that crows were able to colonize the entire globe very quickly not only because they had an exceptional capacity to reach distant locations but also a remarkable ability to persist in suboptimal environments and adapt quickly to new conditions. Beyond the specifics of this case study, our findings more generally suggest that rapid global radiations can be better understood as processes in which dispersal synergises with traits that, like cognition, facilitate survival in suboptimal habitats and ultimately promote the expansion of ecological niches.

## Methods

### Phylogenetic analyses

We computed a new phylogeny of the superfamily Corvoidea, the parent clade containing the family Corvidae. Working at this large phylogenetic scale allowed us to use multiple calibration points distributed across the superfamily (external to Corvidae) and allowed us to compare the diversification rates computed for *Corvus* and Corvidae, with the background rates in their ancestral clade. To build the phylogeny we used the gene supermatrix provided by Jønsson et al.^21^ and updated all missing species in the superfamily with new data from GenBank (GenBank was accessed in late 2017, Supplementary Data File 1). The final dataset included eight nuclear (c-mos, Fib-5, GAPDH, Myo2, ODC, RAG-1, RAG-2 and TGFb2) and four mitochondrial (COI, cytb, ND2 and ND3) genes. All genes were aligned through a translation alignment algorithm implemented in TranslatorX^83^ (http://translatorx.co.uk). Phylogenetic analyses were conducted with the package BEAST v2.4.8^84^ based on an uncorrelated log-normal relaxed clock and a “Yule process” tree prior. We used the same calibrations used in^21^ to estimate our phylogeny in units of time. The best nucleotide substitution model and partition strategy was estimated through a reversible-jump algorithm^85^, as implemented in the plugin RB in the package BEAST. Analyses relied on two independent runs of 100 million generations each. After visualising the traces of both runs in the programme tracer v1.6^86^, and assessing good mixing and convergence of both MCMC chains, we discarded an appropriate 'burn-in' fraction before combining posterior estimates (75,000,000 generations in run 1 and 30,000,000 generations in run 2). We used the same calibrations used in^21^ to estimate our phylogeny in units of time. We calculated the MCC tree with median node heights using TreeAnnotator (also included in BEAST package), setting the posterior probability limit at 0.5. Eleven species (four species for which we had morphological data and seven species for which we had climatic data) and that were not available in GenBank were added manually to the MCC tree and to all trees in our posterior following taxonomic and biogeographic criteria. We performed a sensitivity analysis to assess the effect of the added species in our results (see Supplementary Information File for details on taxon allocation and results of the sensitivity analyses).

### Dynamics of species diversification

We used BAMM v2.5.0^26^ to infer species diversification rates on the MCC tree of the superfamily Corvoidea (only containing species available in GenBank). This analysis was based on two independent rjMCMC, each reliant on four chains with a thinning interval of 10,000 generations and a total chain length of 30 million generations. Prior settings were generated in the R package BAMMtools v2.1.7^44^. After discarding the first 10% of generations as 'burn-in', we assessed the convergence of the MCMCs by visualising the traces of both runs and computing the potential scale reduction factors (ensuring that they approached to 1 and the effective sample sizes of each parameter (ensuring that they were above 500). Species diversification rates across the phylogeny were interpreted by visualising the means of the marginal posterior density of the rates estimated for each branch. We also visualised and interpreted the best-supported shift configurations (i.e. frequencies ≥0.2 in the 95% credible shift set) on the MCC tree and performed a macroevolutionary cohort analysis that estimated the pairwise probability that any two tips in the phylogeny shared the same diversification rate^28^. Finally, we computed and visualised the median diversification rate through time. To integrate phylogenetic uncertainty into our BAMM analyses, we ran a single rjMCMC chain of 30 million generations across a sample of 100 tree topologies randomly selected from the BEAST posterior distribution (posterior set of trees). For each chain, we extracted the mean marginal densities of rates for all tips and compared them across the 100 trees. All BAMM outputs were analysed using the R package BAMMtools^44^.

Aside from BAMM, we also ran MEDUSA in the R package geiger v2.0.6.4^87^ to evaluate birth-death and Yule models while setting shifts between diversification regimes at both nodes and stems. Finally, we used the function fit_hbd_psr_on_grid (R package Castor v1.6.7^88^) to fit PSR in homogenous birth-death models on a time grid (evaluated at 8 points) when investigating variation in diversification dynamics over time in the family Corvidae. Lower and upper bounds for PSR were set to 0 and 5 respectively.

### Phenotypic data collection, morphospace generation and dynamics of phenotypic diversification

The use of osteological specimens allowed us to include three species of extinct *Corvus* in the study (*Corvus moriorum* from Chatham Island/New Zealand and *Corvus impluviatus* and *Corvus viriosus* from Hawaii). We acquired linear measurements in 93 species (237 specimens, Supplementary Data File 2) and geometric morphometrics of beak shape in 96 species (213 specimens, Supplementary Data File 3). All measurements were obtained from high definition photographs taken from standardised positions, using the software ImageJ v1.52^89^. To describe beak shape in the geometric morphometric approach, we placed landmarks at the mid-point of the craniofacial hinge (landmark 1), at the lower margin at the level of the maximum curvature at the rostral end of the *fossa et fenestra antorbitalis* (landmark 2) and at the tip of the beak (landmark 3). These were complemented by 11 equally spaced semilandmarks between landmarks 1 and 3, and by nine equally spaced semilandmarks between landmarks 2 and 3 (Supplementary Fig. 27). Within each species, the landmark coordinates of all specimens were superimposed by means of a Generalised Procrustes analysis^90^, where the position of semilandmarks was optimised by allowing them to slide along their respective curves to minimise bending energy. Superimposed coordinates were then projected to a tangent space, from which we calculated the mean shape per species to be used for all downstream analyses. All the aforementioned GM procedures were performed using the function *gpagen* in the package geomorph v3.2.1^91,92^. In addition to the morphological data specifically collected for this study, we also collated wing shape data (HWI) from a published source^43^.

We used PCAs to generate morphospaces for both linear and geometric morphometric datasets. Morphospaces were computed in the R package mvMORPH v1.1.1^36^ by calculating the covariance matrices of our datasets through the mvgls function, using the rotation-invariant 'ridge quadratic null' penalty, and accounting for intra-specific variation and measurement errors in the model fit (i.e. setting the option SE to TRUE). Given that mis-specifying the evolutionary model can lead to erroneous inferences in phylogenetic PCA^37^, we fitted three different models and used the best fitting one for downstream analyses. The models considered were Brownian motion (BM, in which evolutionary rates are constant and the mean expected trait change is zero), Early Burst (EB, a variant of the BM model in which rates decrease over time) and Ornstein–Uhlenbeck (OU, evolutionary rates are constant, but traits are pulled towards a single optimum value)^93^. The relative fit of these alternatives was assessed through the generalised information criterion (GIC) using the GIC function also in the package mvMORPH. The covariance matrix obtained by the best model was then converted to a correlation matrix and used to estimate the PCA with the function mvgls.pca (also included in mvMORPH). To interpret shape variation along the three first PC axes (see results), we predicted the landmark configurations at the extremes of each PC axes by means of the code provided in^36^ (see function in Supplementary Software). Predicted shapes at PC extremes were compared to the global mean shape by means of a thin-plate spline deformation grid using the function plotRefToTarget from the package geomorph^92^. Additionally, beak shape disparities were compared among Corvidae using α-convex hulls computed through the function ahull from the R package alphahull v2.1^94^.

Beak sizes were estimated by means of centroid sizes, defined as the square root of the sum of the squared distances between the centre of the landmark configuration and each landmark^95^. We applied a PL-MANOVA^96^ of centroid size against landmarks of species´ mean shape configurations (with functions mvgls and manova.gls from the package mvMORPH), using a Pillai test, an EB model and 1000 permutations, to test for the existence of shape-size allometry across species^97^. To address this issue, we used the function mvgls to obtain a size-free correlation matrix that was subsequently used to estimate the phylogenetic PCA as described before. All PCA plots were visualised by means of the function phylomorphospace^98^ (from the R package phytools 0.7–70^99^) to facilitate the phylogenetic interpretation of shape variation.

We used BAMM with identical model settings as those described in our lineage diversification analyses, to explore the evolutionary dynamics of Corvid morphology based on the first three PC axes of phenotypic variation (in both linear and geometric morphometric data). We assessed rate heterogeneity across the MCC tree and the posterior set of trees by means of the same approaches described earlier for species diversification.

We also analysed morphological data with the function transformPhylo.ML from the package motmot v2.1.3^45^, setting the minimum clade size to infer a rate shift at five species and the maximum of rate regimes in the phylogeny at four. We implemented BROWNIE^46^ through the function brownie.lite in phytools 0.7–70^99^. For each tree in our posterior set, we fitted two alternative models of evolution: one assuming a single rate parameter across the entire phylogeny and another one assuming independent rates for the *Corvus* clade (including stem) and the remaining Corvidae ('noncensored' model in ref. ^46^). The two-rates models were fitted by assigning branches to each of the target clades with the function paintSubTree (also in phytools 0.7–70^99^). Model support was evaluated through a likelihood-ratio test against the χ^2^ distribution^46^. The mean subclade disparity through time (DTT) of phenotypic PCs was computed with the function dtt (package Geiger^87^), using average squared Euclidean distances. We compared observed DTT patterns against two sets of simulations, one assuming a single BM rate across the entire tree and the other assuming the rate heterogeneity estimated by BAMM (with accelerated rates within *Corvus*, see function in Supplementary Software). Simulations were based on 1000 replicates after which we computed 95% CI.

### Geographic and climatic diversification

To compare the geographic expansion of *Corvus* with the rest of the genera in Corvidae, we generated presence-absence matrices at a resolution of 0.1° by 0.1° for each corvid genera based on shapefiles provided by BirdLife International (downloaded in Feb 2019^100^). With these, we first plotted and compared geographic distributions and areas among corvid genera as well as between *Corvus* and other Corvidae using a Wagner IV equal-area projection. To explore climatic diversification in Corvidae, we downloaded all observations of species in the family from eBird^49^, following the criteria described in Callaghan et al^101^. and restricting our search from January 2010 to May 2020 (more than 30 million observational records). To reduce redundancy in these data and to avoid potential sampling-related and geographical biases, we subsampled the set of observations to a single locality per species per cell at an approximate resolution of 11 km × 11 km. All species distributions were subsequently visualised to filter out gross errors, such as localities in atypical continents or within water bodies. We then extracted the climatic variables associated with each georeferenced observation using the function 'extract' in the package raster v3.4-10^102^ and climate data from WorldClim v2.1^103^ at a resolution of 1.5 min. The PCA of the background climatic space was produced from the combined dataset for all corvids using the function dudi.pca in R package ade4 v1.7-16a^104^. For broad-level comparisons we approximated the position of each species in climatic niche space as their mean values in the first two PCA axes. Climatic variation across species was visualised through the function phylomorphospace^98^ in phytools v0.7–70^99^ to facilitate phylogenetic interpretation. Finally, we also used our curated eBird dataset to estimate the number of unique sympatric assemblages of congeneric species in each genus. A proxy for the levels of potential competition among closely related species (see function in Supplementary Software).

### Dynamics of relative brain size evolution

We measured brain volumes of 76 species (197 specimens) of Corvids, by filling the brain cavity of skulls with 1 mm glass microballoons (GB 01, conservation resources UK limited) of known density and weighing these microballoons with a digital scale at 0.01 gram precision. We converted weights to volumes using their known density and obtained relative brain volumes (Supplementary Data File 4) by regressing log-transformed absolute mean brain volumes against log-transformed mean femur lengths. We chose femur length as a proxy for body size because this metric is readily available for all species (including extinct ones) and it is the osteological measure that more closely covaries with body fresh weight in Corvidae (*n* = 19, cor = 0.9) (Supplementary Fig. 28).

We used phylogenetic linear regression models in the function phylolm (available in the R package phylolm v2.6.2^105^) to compute residual brain sizes, under four different models of evolution: BM, OUrandomRoot (OU process with a stationary distribution for the ancestral state at the root) and OUfixedRoot (OU process with an estimated ancestral state at the root) and EB (early burst). Additionally, we fitted one more phylolm model using Pagel’s lambda (a weighing parameter that estimates the extent to which tip similarities can be explained by a BM process). Residual brain sizes for downstream analyses were extracted from the best fitting model given AIC scores^106^ (i.e. the model with Pagel’s lambda). We note that as expected, brain residuals are uncorrelated with body size (phylogenetic regression: *F* = −0.01, *p* value = 0.89). We also used phylolm to test for relative brain size differences between *Corvus* and the rest of the family Corvidae (phylogenetic ANOVA), comparing the supports of all previously described models.

The dynamics of relative brain evolution were characterised with BAMM, MOTMOT and BROWNIE using the settings described in earlier sections. To study variation in body size (femur length), HWI and relative brain size through time, we projected the phylogeny into spaces defined by phenotypic values using the function phenogram in the R package phytools^99^. Additionally, we visualised the average ancestral state values of each trait at different time points using the function ace in the package ape v5.5^107^. To better assess the effects of *Corvus* we compared these macroevolutionary patterns with those obtained after removing all species in this genus from the phylogeny and recomputing the averages.

### Reporting summary

Further information on research design is available in the Nature Research Reporting Summary linked to this article.

## Supplementary information

Supplementary Information

Description of Additional Supplementary Files

Supplementary Software

Supplementary Data 1

Supplementary Data 2

Supplementary Data 3

Supplementary Data 4

Reporting Summary

## Data Availability

Morphological data is available as Supplementary Data Files 1–4. Climatic data were available in the WorldClim database (http://worldclim.org). Shapefiles of the distribution of each species of corvid were downloaded from BirdLife International (http://datazone.birdlife.org/species/requestdis). The coordinates of observations of corvid species were obtained from eBird (https://ebird.org/science/use-ebird-data/download-ebird-data-products).

## Code availability

The custom code used in this study is provided in Supplementary Software.
