# Supplementary material for: Niche expansion and adaptive divergence in the global radiation of crows and ravens
Source: Nat Commun. 2022 Apr 21;13:2086. doi: 10.1038/s41467-022-29707-5 (PMC9023458; doi:10.1038/s41467-022-29707-5)
Supplement: Supplementary file 1 — Supplementary Information [file 41467_2022_29707_MOESM1_ESM.pdf]

Supplementary Information for

NICHE EXPANSION AND ADAPTIVE DIVERGENCE IN THE GLOBAL RADIATION OF CROWS AND RAVENS

Joan Garcia-Porta, Daniel Sol, Matt Pennell, Ferran Sayol, Antigoni Kaliontzopoulou & Carlos Botero

Joan Garcia-Porta

Email: j.garcia-porta@wustl.edu

Supplementary note

Taxon allocation and sensitivity analyses. 11 species (three species for which we had morphological data and eight species for which we had climatic data) and that were not available in GenBank (accessed in November 2017) were added manually to the summary tree following taxonomic and biogeographic criteria. For example, given the remoteness of the Hawaiian archipelago, we assumed that all *Corvus* endemics from this archipelago were derived from a single colonization event and that the extinct *C. viriosus* and *C. impluviatus* were monophyletic with *C. hawaiiensis*[1]. Given that non-phylogenetic (preliminary) and phylogenetic PCA analyses showed that *C. viriosus* and *C. impluviatus* are more morphologically similar to each other than to *C. hawaiiensis*, we further assumed a phylogenetic structure of (*C. hawaiiensis* (*C. viriosus*, *C.*

impluviatus))), with a deep split at 3 Ma, which is the maximum estimated age of O'ahu, the oldest island containing fossils of *C. viriosus*[1,2]. All of the other species that were not available in GenBank (i.e., *Podoces panderi, Podoces pleskei, Urocissa whiteheadi, Urocissa ornata, Dendrocitta leucogastra, Dendrocitta occipitalis, Dendrocitta bayleii, Crypsirina cucullata* and *Platysmurus aterrimus*) were randomly assigned to their respective genera with random branch lengths to the maximum credibility tree and to each tree topology in the posterior.

All but one of the findings presented in the main text are robust to these taxonomic additions (Supplementary Figure 29-32). Specifically, the only observed exception is that when *C. viriosus* and *C. impluviatus* are excluded from the phylogeny, the detected acceleration in beak shape component GM2 is no longer general to all *Corvus* and is rather restricted to a subclade within it (Supplementary Figure 30). Although this minor difference does not alter the general conclusions presented in the main text in any way, we note that a *Corvus*-wide acceleration is more likely in our opinion because the phylogenetic placement and branch length used to add these two species to the phylogeny are extremely well-supported by evidence. Furthermore, given that the age of O'ahu sets an upper limit to the deepest split in this Hawaiian clade (3 Ma), we also note that branch length uncertainty is one-tailed and that our estimate of *Corvus*-wide divergence in GM2 could consequently only increase if these species happened to have diverged more recently.

Supplementary figures

Supplementary Figure 1

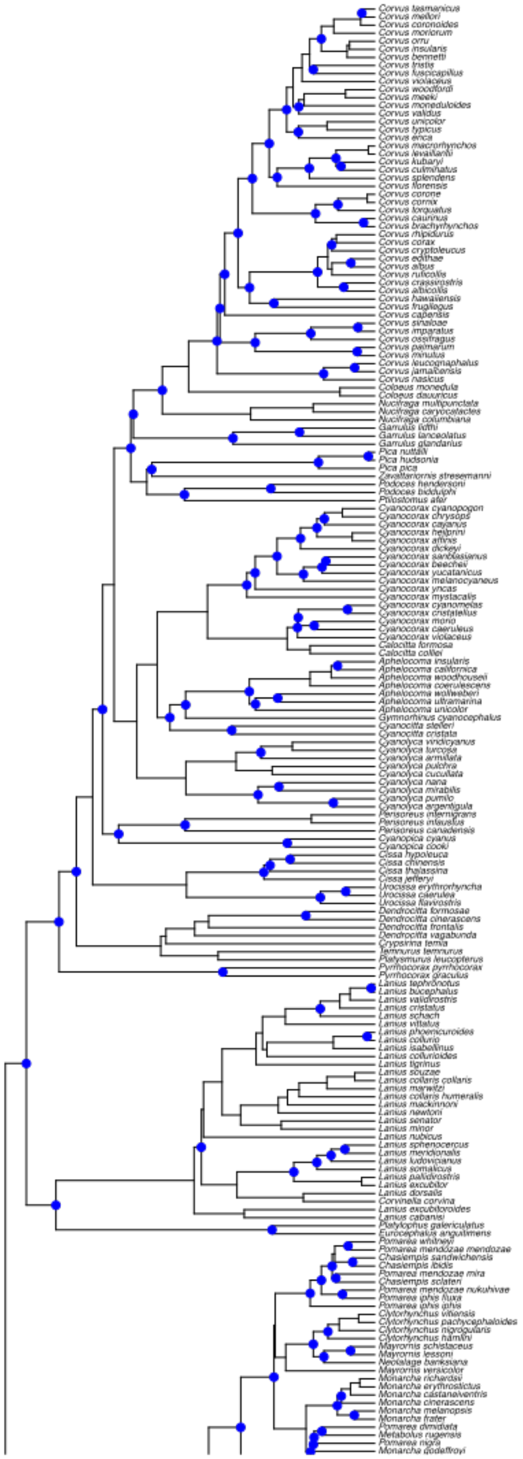

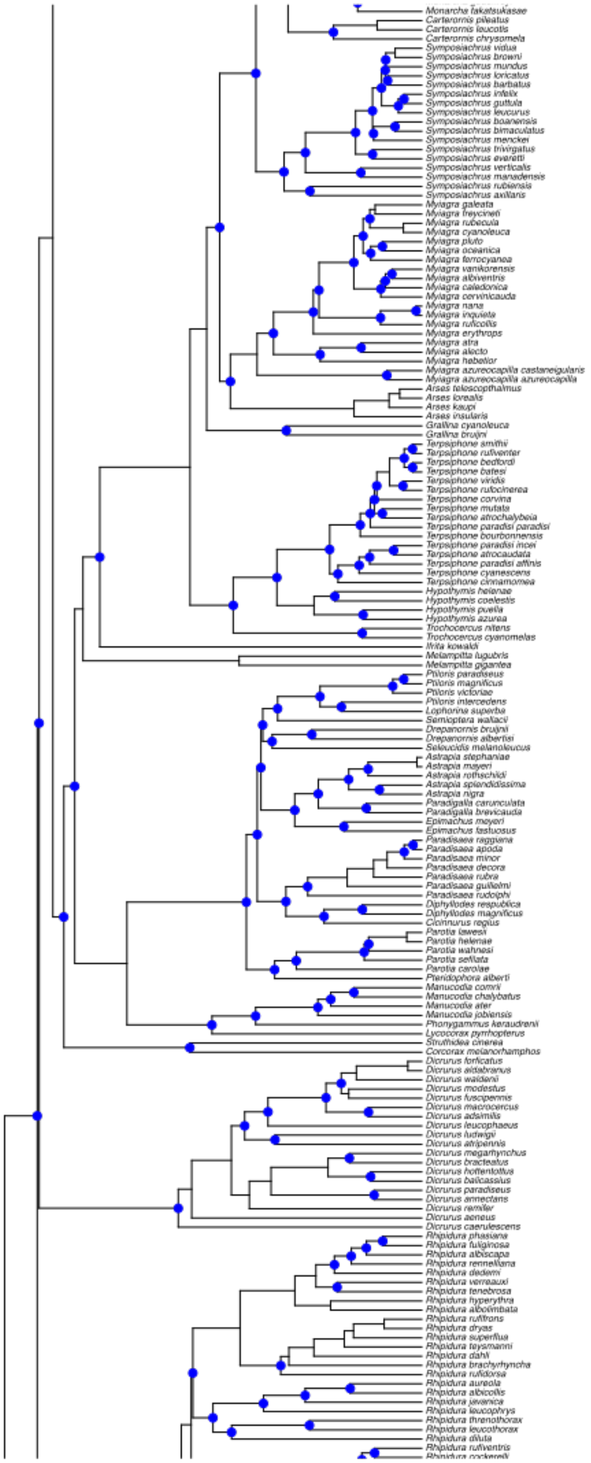
Monarcha takatsukasae
Carterornis pileatus
Carterornis leucotis
Carterornis chrysomela
Symposiachrus vidua
Symposiachrus browni
Symposiachrus mundus
Symposiachrus loricatus
Symposiachrus barbatus
Symposiachrus infelix
Symposiachrus guttula
Symposiachrus leucurus
Symposiachrus boanensis
Symposiachrus bimaculatus
Symposiachrus menckei
Symposiachrus trivirgatus
Symposiachrus everetti
Symposiachrus verticalis
Symposiachrus manadensis
Symposiachrus rubiensis
Symposiachrus axillaris
Myiagra galeata
Myiagra freycineti
Myiagra rubecula
Myiagra cyanoleuca
Myiagra pluto
Myiagra oceanica
Myiagra ferrocyanea
Myiagra vanikorensis
Myiagra albiventris
Myiagra caledonica
Myiagra cervinicauda
Myiagra nana
Myiagra inquieta
Myiagra ruficollis
Myiagra erythrops
Myiagra atra
Myiagra alecto
Myiagra hebetior
Myiagra azureocapilla castaneigularis
Myiagra azureocapilla azureocapilla
Arses telescopthalmus
Arses lorealis
Arses kaupi
Arses insularis
Grallina cyanoleuca
Grallina bruijni
Terpsiphone smithii
Terpsiphone rufiventer
Terpsiphone bedfordi
Terpsiphone batesi
Terpsiphone viridis
Terpsiphone rufocinerea
Terpsiphone corvina
Terpsiphone mutata
Terpsiphone atrochalybeia
Terpsiphone paradisi paradisi
Terpsiphone bourbonnensis
Terpsiphone paradisi incei
Terpsiphone atrocaudata
Terpsiphone paradisi affinis
Terpsiphone cyanescens
Terpsiphone cinnamomea
Hypothymis helenae
Hypothymis coelestis
Hypothymis puella
Hypothymis azurea
Trochocercus nitens
Trochocercus cyanomelas
Ifrita kowaldi
Melampitta lugubris
Melampitta gigantea
Ptiloris paradiseus
Ptiloris magnificus
Ptiloris victoriae
Ptiloris intercedens
Lophorina superba
Semioptera wallacii
Drepanornis bruijnii
Drepanornis albertisi
Seleucidis melanoleucus
Astrapia stephaniae
Astrapia mayeri
Astrapia rothschildi
Astrapia splendidissima
Astrapia nigra
Paradigalla carunculata
Paradigalla brevicauda
Epimachus meyeri
Epimachus fastuosus
Paradisaea raggiana
Paradisaea apoda
Paradisaea minor
Paradisaea decora
Paradisaea rubra
Paradisaea guilielmi
Paradisaea rudolphi
Diphyllodes respublica
Diphyllodes magnificus
Cicinnurus regius
Parotia lawesii
Parotia helenae
Parotia wahnesi
Parotia sefilata
Parotia carolae
Pteridophora alberti
Manucodia comrii
Manucodia chalybatus
Manucodia ater
Manucodia jobiensis
Phonygammus keraudrenii
Lycocorax pyrrhopterus
Struthidea cinerea
Corcorax melanorhamphos
Dicrurus forficatus
Dicrurus aldabranus
Dicrurus waldenii
Dicrurus modestus
Dicrurus fuscipennis
Dicrurus macrocercus
Dicrurus adsimilis
Dicrurus leucophaeus
Dicrurus ludwigii
Dicrurus atripennis
Dicrurus megarhynchus
Dicrurus bracteatus
Dicrurus hottentottus
Dicrurus balicassius
Dicrurus paradiseus
Dicrurus annectans
Dicrurus remifer
Dicrurus aeneus
Dicrurus caerulescens
Rhipidura phasiana
Rhipidura fuliginosa
Rhipidura albiscapa
Rhipidura rennelliana
Rhipidura dedemi
Rhipidura verreauxi
Rhipidura tenebrosa
Rhipidura hyperythra
Rhipidura albolimbata
Rhipidura rufifrons
Rhipidura dryas
Rhipidura superflua
Rhipidura teysmanni
Rhipidura dahli
Rhipidura brachyrhyncha
Rhipidura rufidorsa
Rhipidura aureola
Rhipidura albicollis
Rhipidura javanica
Rhipidura leucophrys
Rhipidura threnothorax
Rhipidura leucothorax
Rhipidura diluta
Rhipidura rufiventris

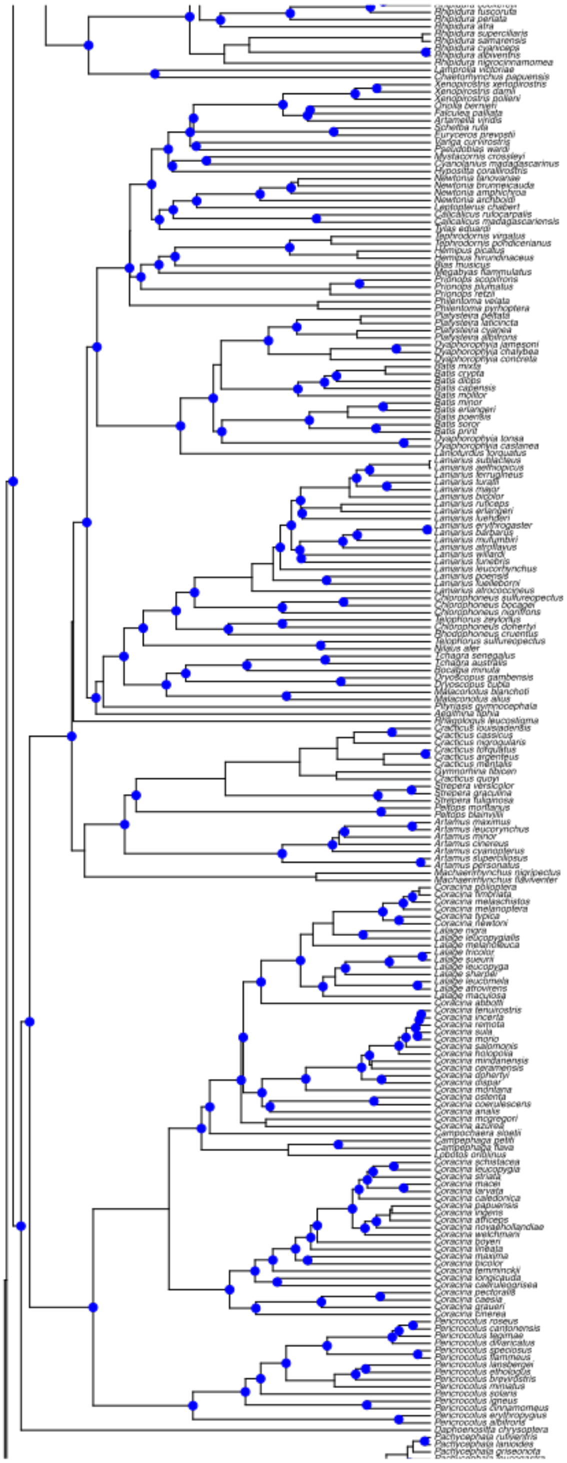
Rhipidura fuscorufa
Rhipidura perlata
Rhipidura atra
Rhipidura superciliaris
Rhipidura samarensis
Rhipidura cyaniceps
Rhipidura albiventris
Rhipidura nigrocinnamomea
Lamprolia victoriae
Chaetorhynchus papuensis
Xenopirostris xenopirostris
Xenopirostris damii
Xenopirostris polleni
Oriolia bernieri
Falculea palliata
Artamella viridis
Schetba rufa
Euryceros prevostii
Vanga curvirostris
Pseudobias wardi
Mystacornis crossleyi
Cyanolanius madagascarinus
Hypositta corallirostris
Newtonia fanovanae
Newtonia brunneicauda
Newtonia amphichroa
Newtonia archboldi
Leptopterus chabert
Calicalicus rufocarpalis
Calicalicus madagascariensis
Tylas eduardi
Tephrodornis virgatus
Tephrodornis pondicerianus
Hemipus picatus
Hemipus hirundinaceus
Bias musicus
Megabyas flammulatus
Prionops scopifrons
Prionops plumatus
Prionops retzii
Philentoma velata
Philentoma pyrhoptera
Platysteira peltata
Platysteira laticincta
Platysteira cyanea
Platysteira albifrons
Dyaphorophyia jamesoni
Dyaphorophyia chalybea
Dyaphorophyia concreta
Batis mixta
Batis crypta
Batis diops
Batis capensis
Batis molitor
Batis minor
Batis erlangeri
Batis poensis
Batis soror
Batis pririt
Dyaphorophyia tonsa
Dyaphorophyia castanea
Lanioturdus torquatus
Laniarius sublacteus
Laniarius aethiopicus
Laniarius ferrugineus
Laniarius turatii
Laniarius major
Laniarius bicolor
Laniarius ruficeps
Laniarius erlangeri
Laniarius luehderi
Laniarius erythrogaster
Laniarius barbarus
Laniarius mufumbiri
Laniarius atroflavus
Laniarius willardi
Laniarius funebris
Laniarius leucorhynchus
Laniarius poensis
Laniarius fuelleborni
Laniarius atrococcineus
Chlorophoneus sulfureopectus
Chlorophoneus bocagei
Chlorophoneus nigrifrons
Telophorus zeylonus
Chlorophoneus dohertyi
Rhodophoneus cruentus
Telophorus sulfureopectus
Nilaus afer
Tchagra senegalus
Tchagra australis
Bocagia minuta
Dryoscopus gambensis
Dryoscopus cubla
Malaconotus blanchoti
Malaconotus alius
Pityriasis gymnocephala
Aegithina tiphia
Rhagologus leucostigma
Cracticus louisiadensis
Cracticus cassicus
Cracticus nigrogularis
Cracticus torquatus
Cracticus argenteus
Cracticus mentalis
Gymnorhina tibicen
Cracticus quoyi
Strepera versicolor
Strepera graculina
Strepera fuliginosa
Peltops montanus
Peltops blainvillii
Artamus maximus
Artamus leucorynchus
Artamus minor
Artamus cinereus
Artamus cyanopterus
Artamus superciliosus
Artamus personatus
Machaerirhynchus nigripectus
Machaerirhynchus flaviventer
Coracina polioptera
Coracina fimbriata
Coracina melaschistos
Coracina melanoptera
Coracina typica
Coracina newtoni
Lalage nigra
Lalage leucopygialis
Lalage melanoleuca
Lalage tricolor
Lalage sueurii
Lalage leucopyga
Lalage sharpei
Lalage leucomela
Lalage atrovirens
Lalage maculosa
Coracina abbotti
Coracina tenuirostris
Coracina incerta
Coracina remota
Coracina sula
Coracina morio
Coracina salomonis
Coracina holopolia
Coracina mindanensis
Coracina ceramensis
Coracina dohertyi
Coracina dispar
Coracina montana
Coracina ostenta
Coracina coerulescens
Coracina analis
Coracina mcgregori
Coracina azurea
Campochaera sloetii
Campephaga petiti
Campephaga flava
Lobotos oriolinus
Coracina schistacea
Coracina leucopygia
Coracina striata
Coracina maceí
Coracina larvata
Coracina caledonica
Coracina papuensis
Coracina ingens
Coracina atriceps
Coracina novaehollandiae
Coracina welchmani
Coracina boyeri
Coracina lineata
Coracina maxima
Coracina bicolor
Coracina temminckii
Coracina longicauda
Coracina caeruleogrisea
Coracina pectoralis
Coracina caesia
Coracina graueri
Coracina cinerea
Pericrocotus roseus
Pericrocotus cantonensis
Pericrocotus tegimae
Pericrocotus divaricatus
Pericrocotus speciosus
Pericrocotus flammeus
Pericrocotus lansbergei
Pericrocotus ethologus
Pericrocotus brevirostris
Pericrocotus miniatus
Pericrocotus solaris
Pericrocotus igneus
Pericrocotus cinnamomeus
Pericrocotus erythropygius
Pericrocotus albifrons
Daphoenositta chrysoptera
Pachycephala rufiventris
Pachycephala lanioides
Pachycephala griseonota

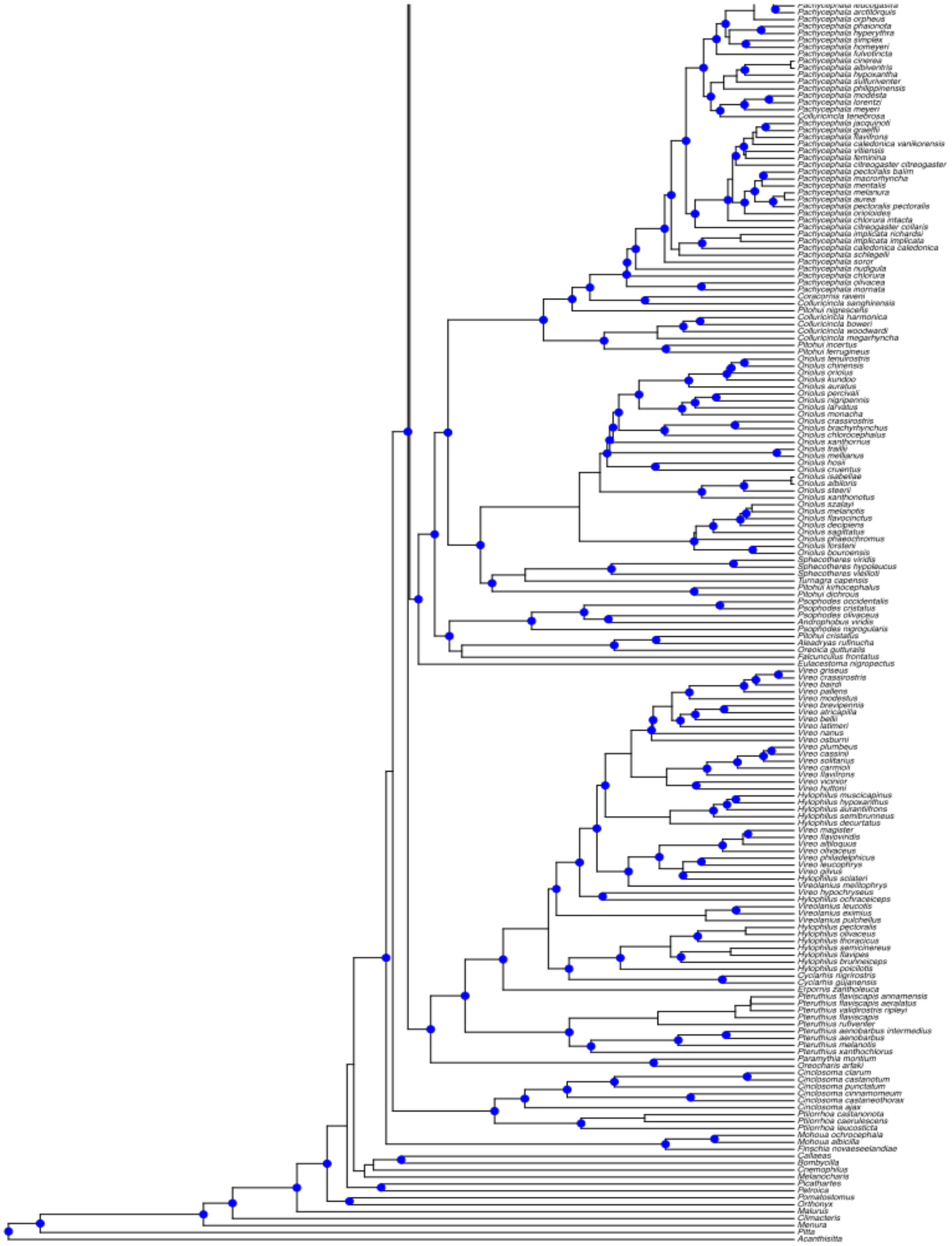

Maximum clade credibility tree of the family Corvoidea as inferred through BEAST. Blue dots depict nodes with > 0.9 of posterior probability (high support).

Supplementary Figure 2

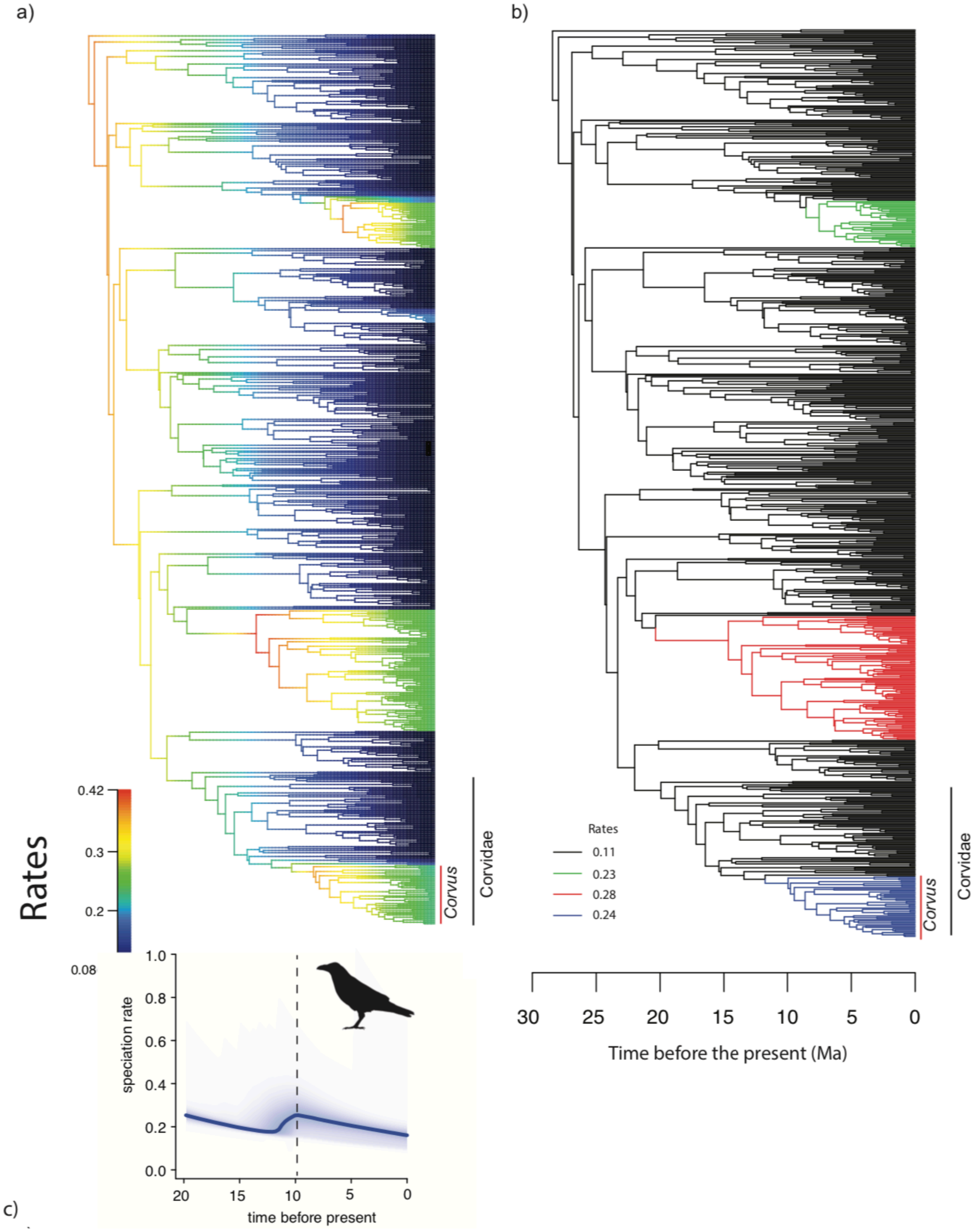

Species diversification rate heterogeneity in the MCC tree of the superfamily Corvoidea. (a) Rates derived from the BAMM analysis. (b) Rates derived from MEDUSA. (c) Species diversification rates through time estimated for the family Corvidae as estimated by BAMM (derived from the analysis on Corvoidea). Shaded areas depict 95% credible intervals.

Supplementary Figure 3

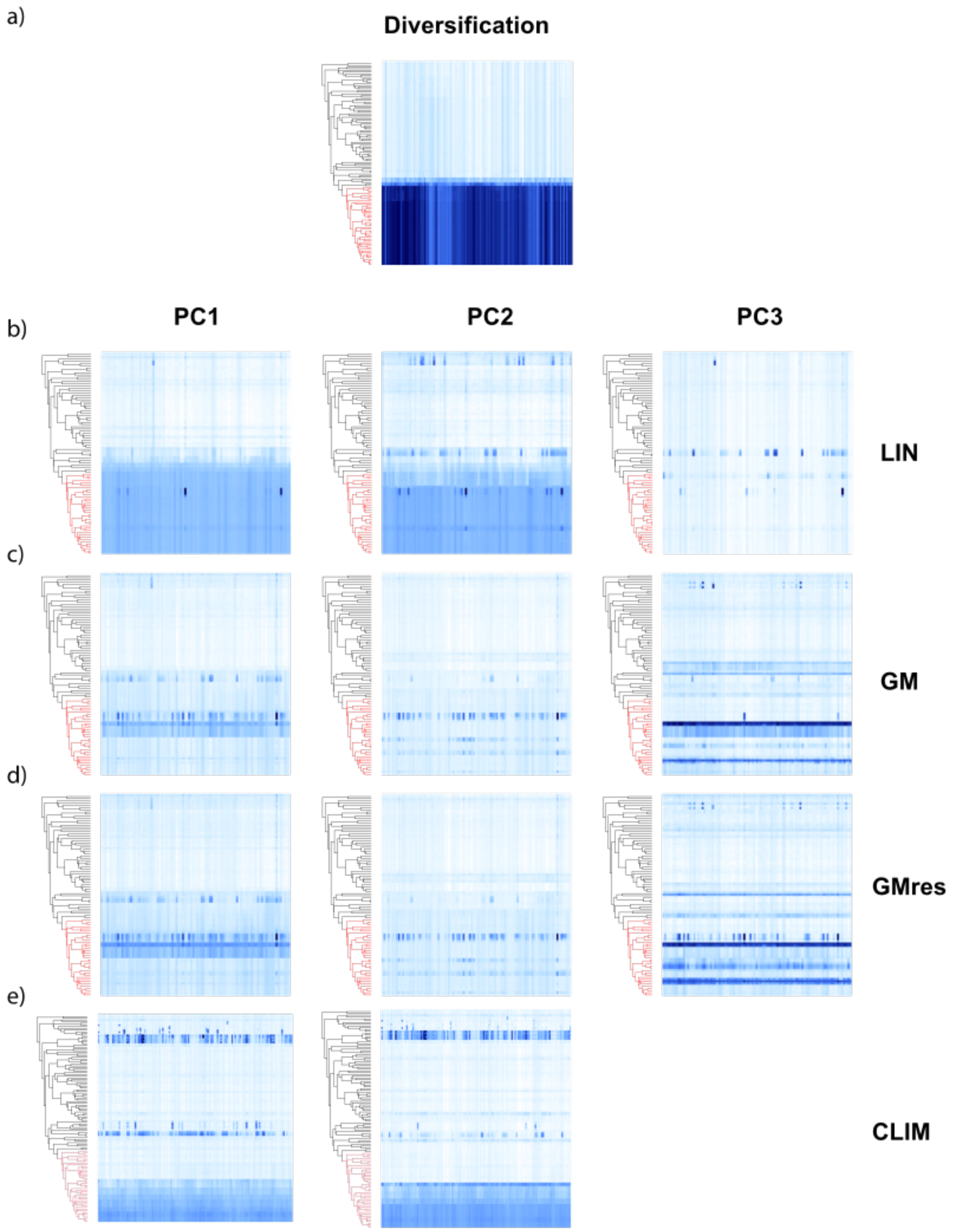

Robustness of our BAMM findings to phylogenetic uncertainty. Visualization of the log-mean tip rate variation across a sample of trees randomly selected from the BEAST posterior distribution. Darker blues depict higher rates. Rows depict our analyses for (a) species diversification, (b) linear dataset (LIN), (c) geometric morphometrics dataset (GM), (d) residuals from the geometric morphometric dataset (GMres) and (e) climatic dataset (CLIM).

Supplementary Figure 4

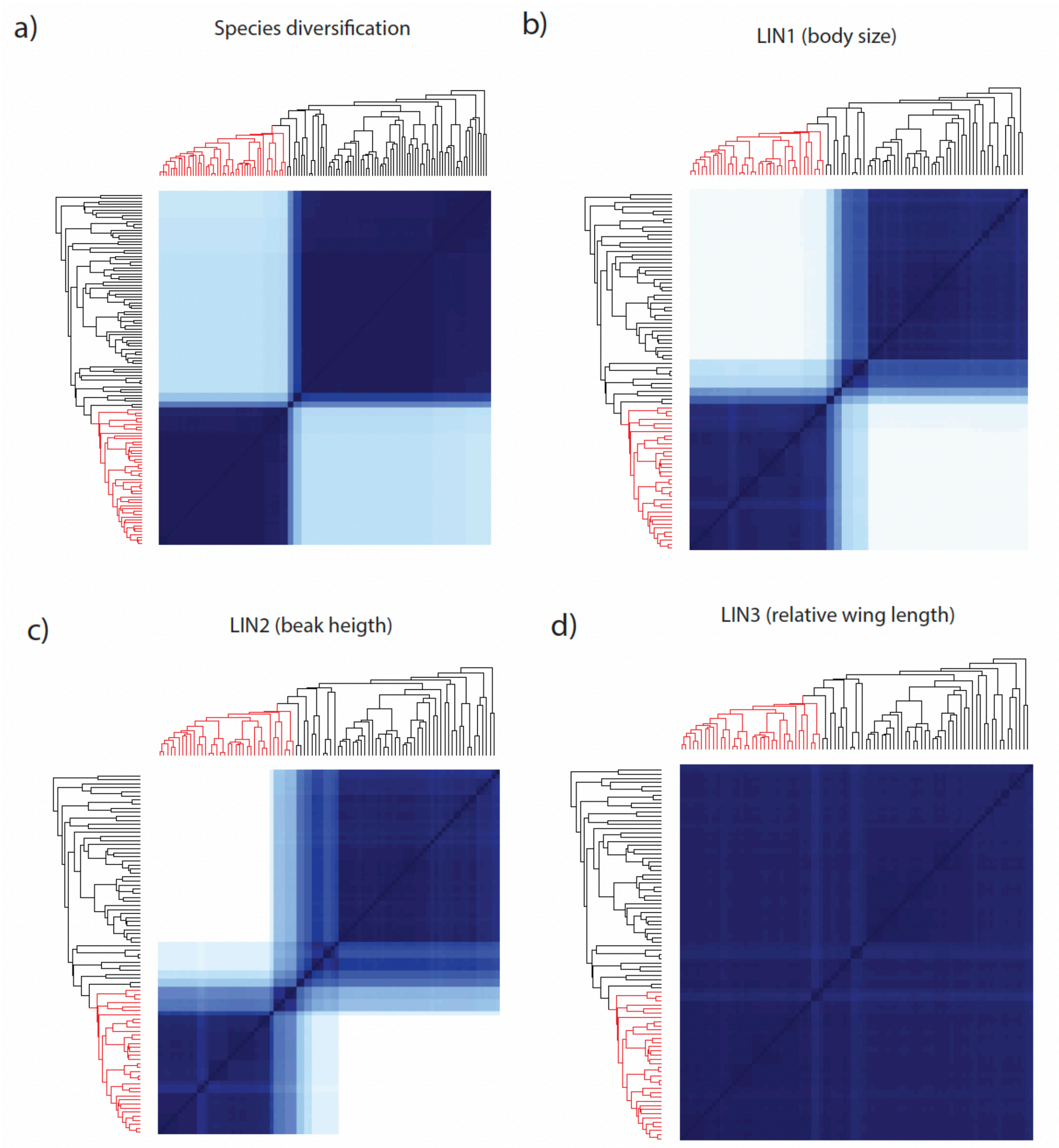

Cohort analyses for species (a) and morphological (linear dataset = LIN, b-d) diversification in Corvidae. The genus *Corvus* is identified in red within each phylogeny. The intensity of blue colouring in the macroevolutionary cohort matrices is proportional to the pairwise probability that two species share a common macroevolutionary rate regime (darker = higher probability).

Supplementary Figure 5

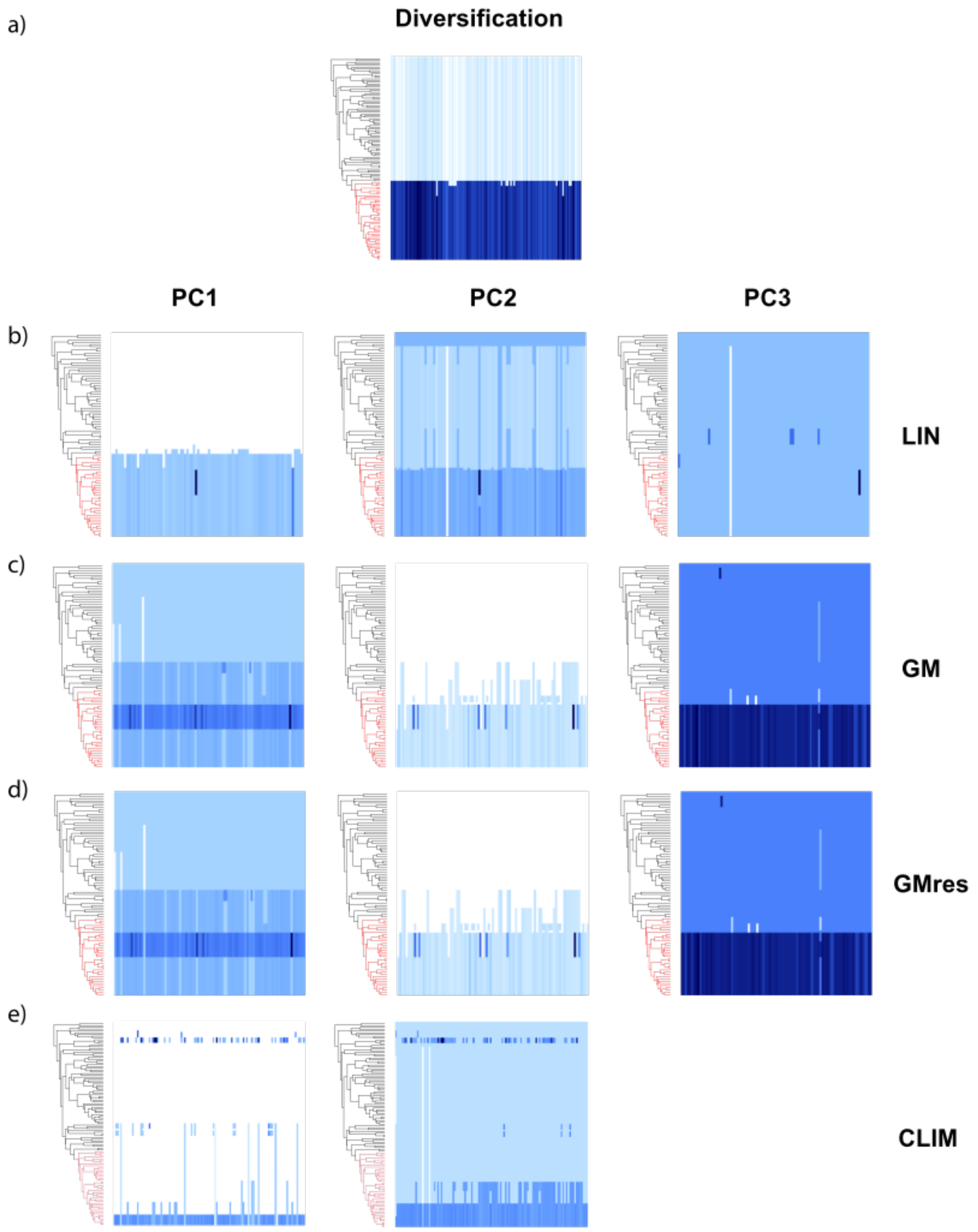

Robustness of our MEDUSA and MOTMOT findings to phylogenetic uncertainty. Visualization of the log-mean tip rate variation across a sample of trees randomly selected from the BEAST posterior distribution. Darker blues depict higher rates. Rows depict our analyses for (a) species diversification (computed in MEDUSA), (b) linear dataset (LIN), (c) geometric morphometrics dataset (GM), (d) residuals from the geometric morphometric dataset (GMres) and (e) climatic dataset (CLIM) (b, c, d, and e computed in MOTMOT).

Supplementary Figure 6

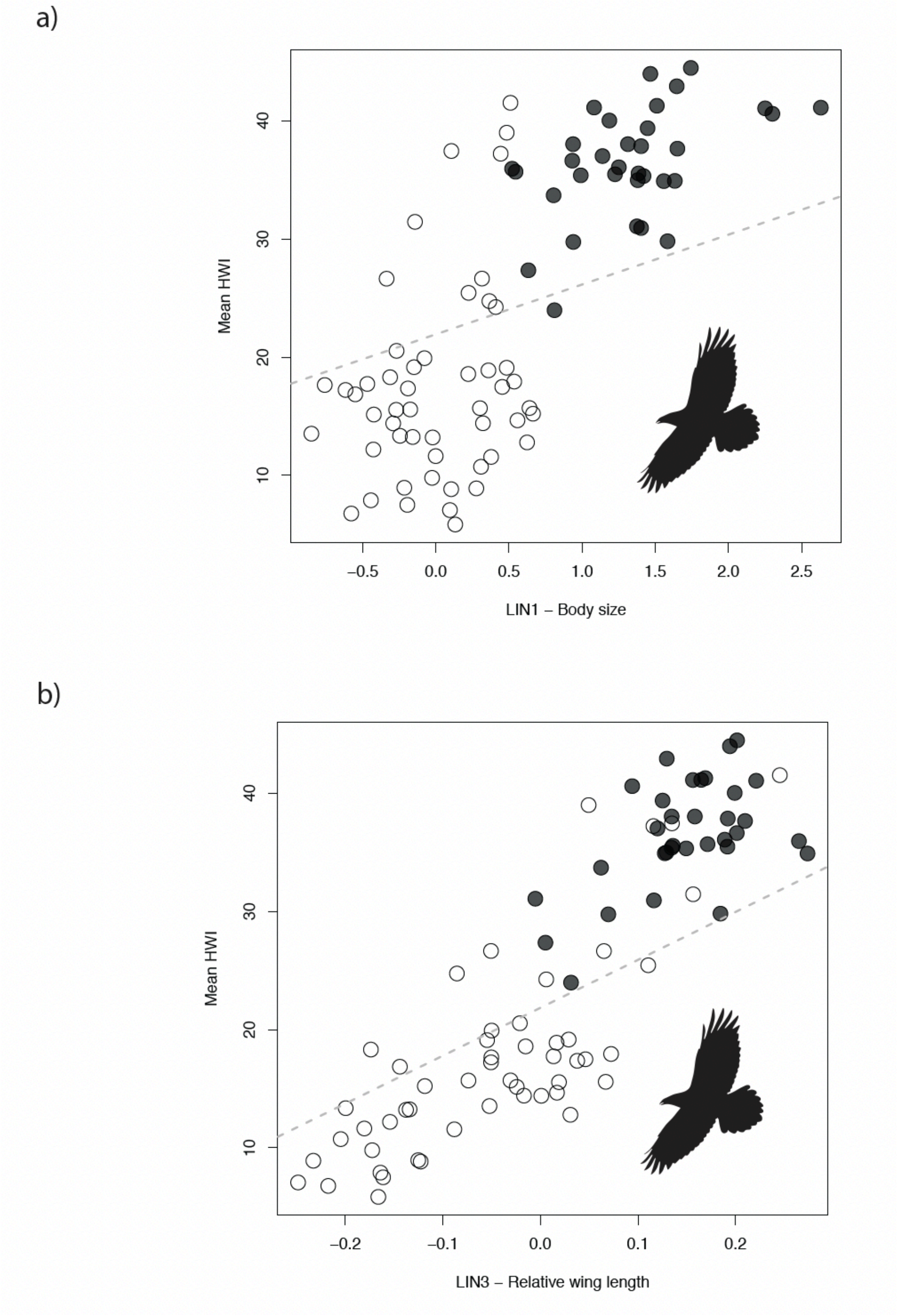

Relationship between body size, relative wing length and dispersal ability among Corvidae. LIN1 (body size) and LIN3 (relative wing length) in our dataset are significantly correlated with mean "hand-wing index" (HWI), a well-known proxy of the capacity for sustained long-distance flight. Species in *Corvus* are depicted in black. Phylogenetic regression lines are depicted in light grey. LIN = linear dataset.

Supplementary Figure 7

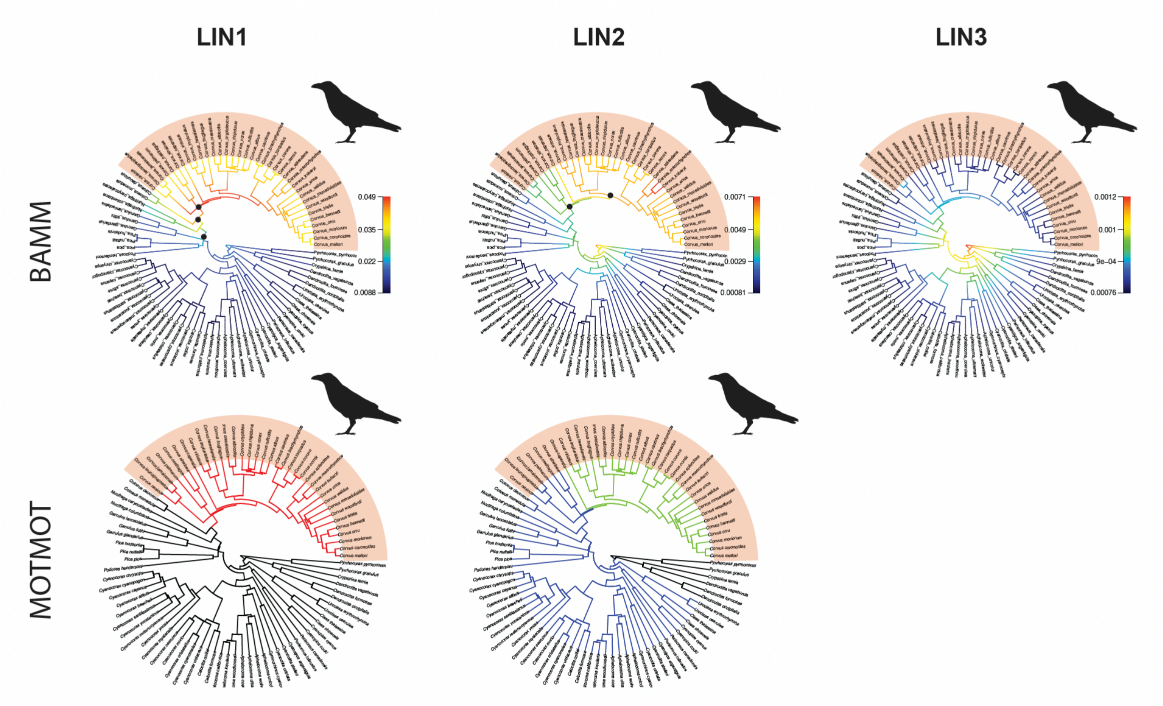

Rates of morphological evolution (Linear dataset - LIN dataset). Rates of morphological evolution as calculated by BAMM are presented in the top row, with colours along branches denoting rates of phenotypic evolution. Black dots indicate the location of the most frequent shifts detected by BAMM (frequency > 0.2). Rate heterogeneities as estimated by MOTMOT are plotted in the bottom row, except for LIN3, for which MOTMOT estimated a single rate. Rates were estimated on the scores of a phylogenetic PCA derived from the linear measurements. In all cases, the *Corvus* clade is highlighted in orange.

Supplementary Figure 8

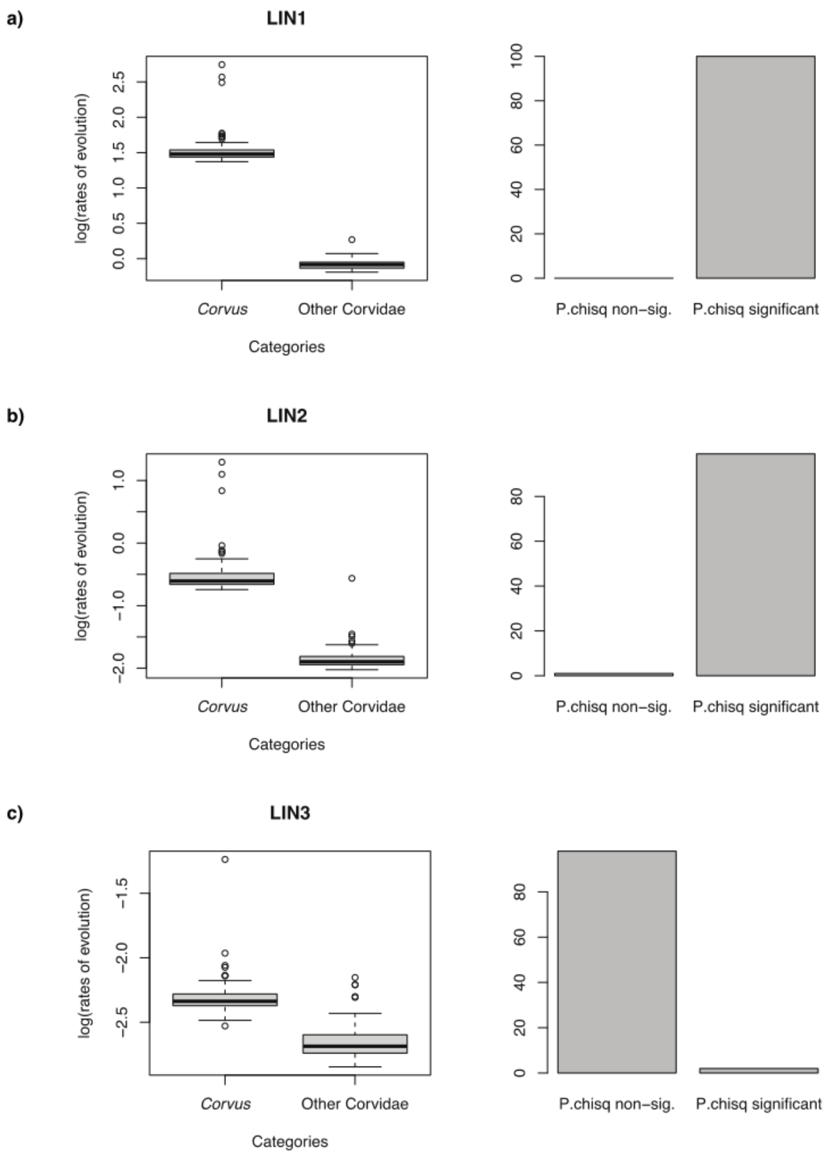

Rates of morphological evolution (Linear dataset - LIN dataset). The left panels show the rates of morphological evolution estimated by fitting, on each of the set posterior trees (100 trees), a Brownian rate variation ("noncensored") model (described in O'Meara et al. 2006; Evolution) that assumes two rates: a rate for the *Corvus* clade (including stem) and a rate for the remaining Corvidae. Box plots indicate median (middle line), 25th, 75th percentile (box), and 5th and 95th percentile (whiskers). The right panels show the proportion of significant p-values (one-tailed, based on 1,000 simulations) in likelihood ratio test against a χ^2 distribution (significant p-values imply rate variation).

Supplementary Figure 9

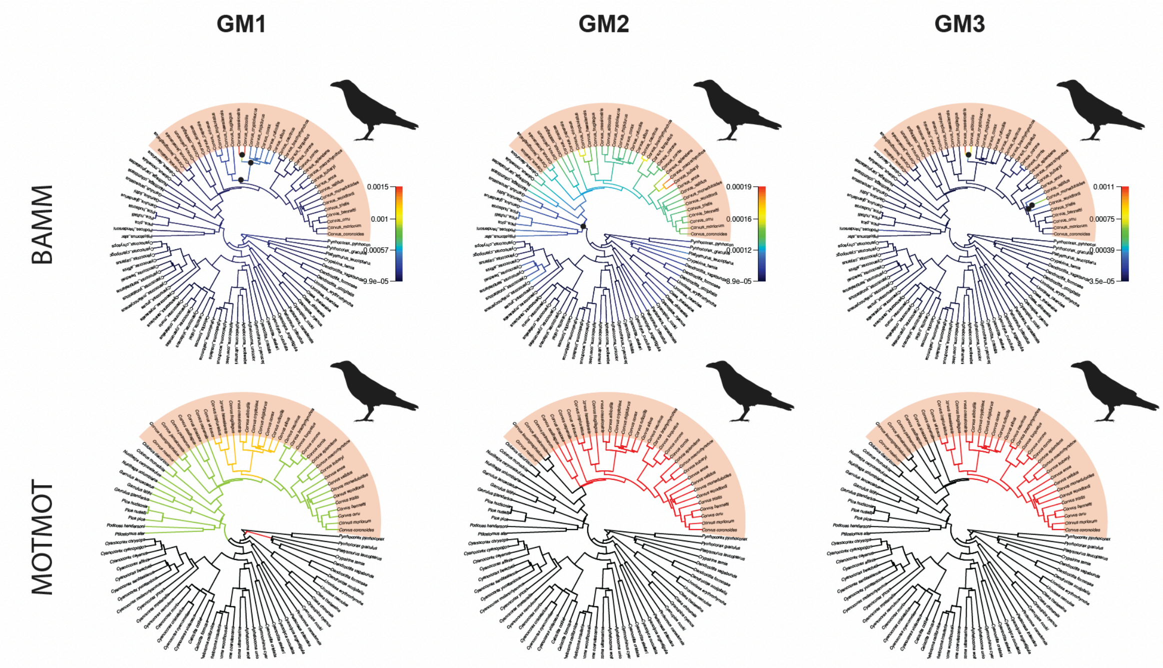

Rates of beak shape evolution (Geometric morphometric dataset - GM dataset). Rates of trait evolution as calculated by BAMM are presented in the top row, with colours along branches denoting instantaneous rates of phenotypic evolution. Dots indicate the locations of most frequent shifts detected by BAMM (frequency > 0.2). Plots of rate heterogeneity as estimated by MOTMOT are presented in the bottom row. Rates were estimated on the scores of a phylogenetic PCA derived from the geometric morphometrics dataset (quantifying beak shape variation). In all cases, the *Corvus* clade is highlighted in orange.

Supplementary Figure 10

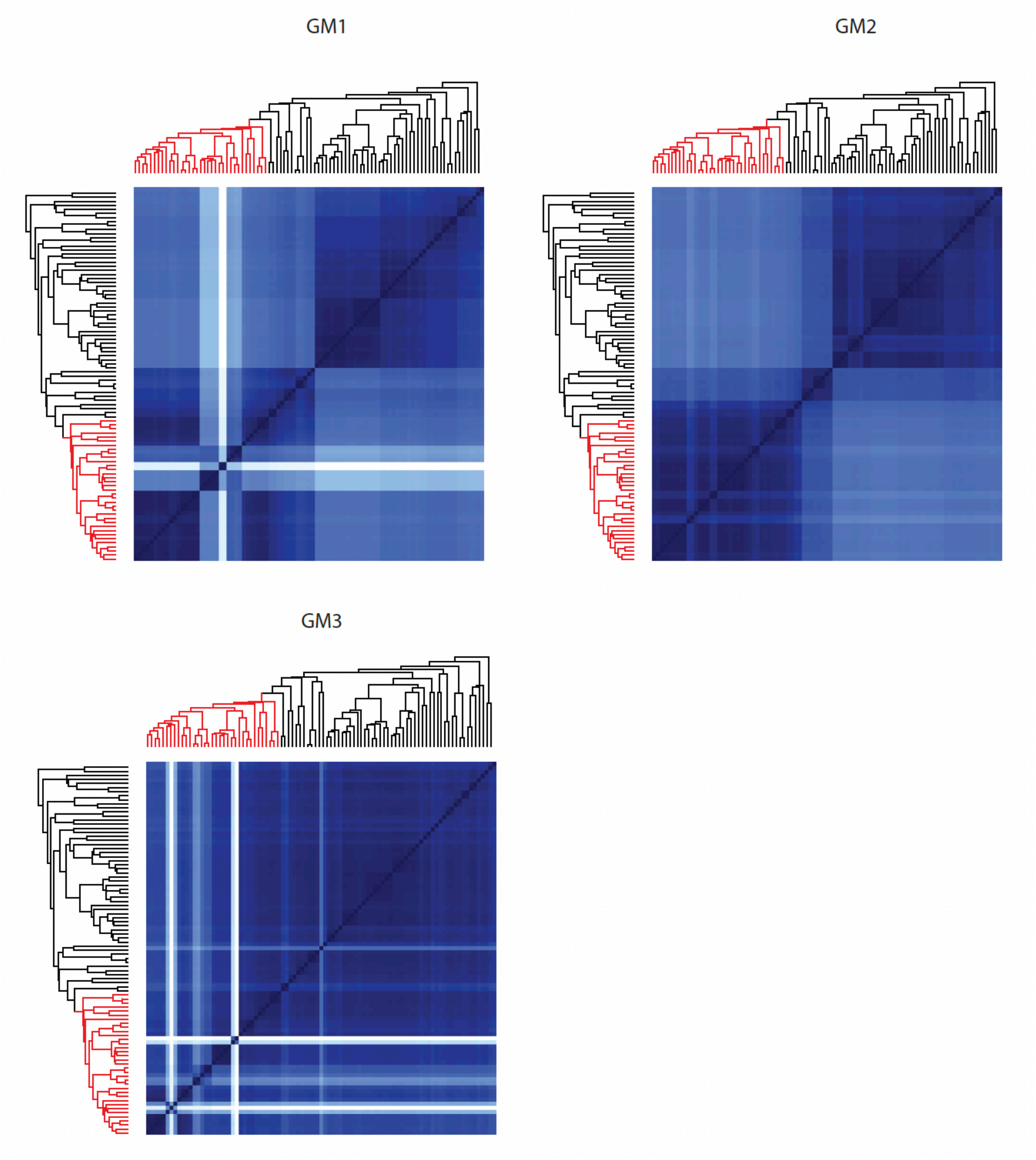

Cohort analyses for beak shape diversification in Corvidae (Geometric morphometric dataset - GM dataset). The genus *Corvus* is identified in red within each phylogeny. The intensity of blue colouring in the macroevolutionary cohort matrices is proportional to the pairwise probability that two species share a common macroevolutionary rate regime (darker = higher probability).

Supplementary Figure 11

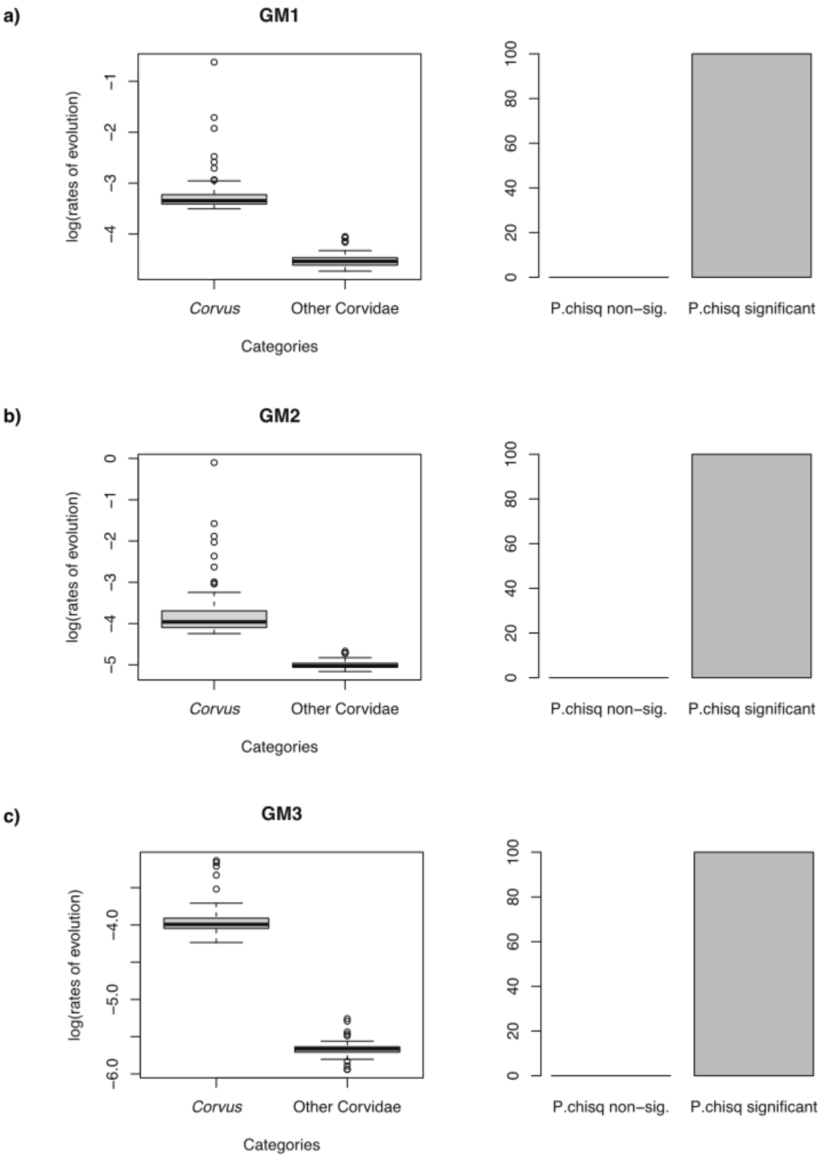

Rates of beak shape evolution (Geometric morphometric dataset - GM dataset). The left panels show the rates of morphological evolution estimated by fitting, on each of the set posterior trees (100 trees), a Brownian rate variation ("noncensored") model (described in O'Meara et al. 2006; Evolution) that assumes two rates: a rate for the *Corvus* clade (including stem) and a rate for the remaining Corvidae. Box plots indicate median (middle line), 25th, 75th percentile (box), and 5th and 95th percentile (whiskers). The right panels show the proportion of significant p-values (one-tailed, based on 1,000 simulations) in likelihood ratio test against a χ^2 distribution (significant p-values imply rate variation).

Supplementary Figure 12

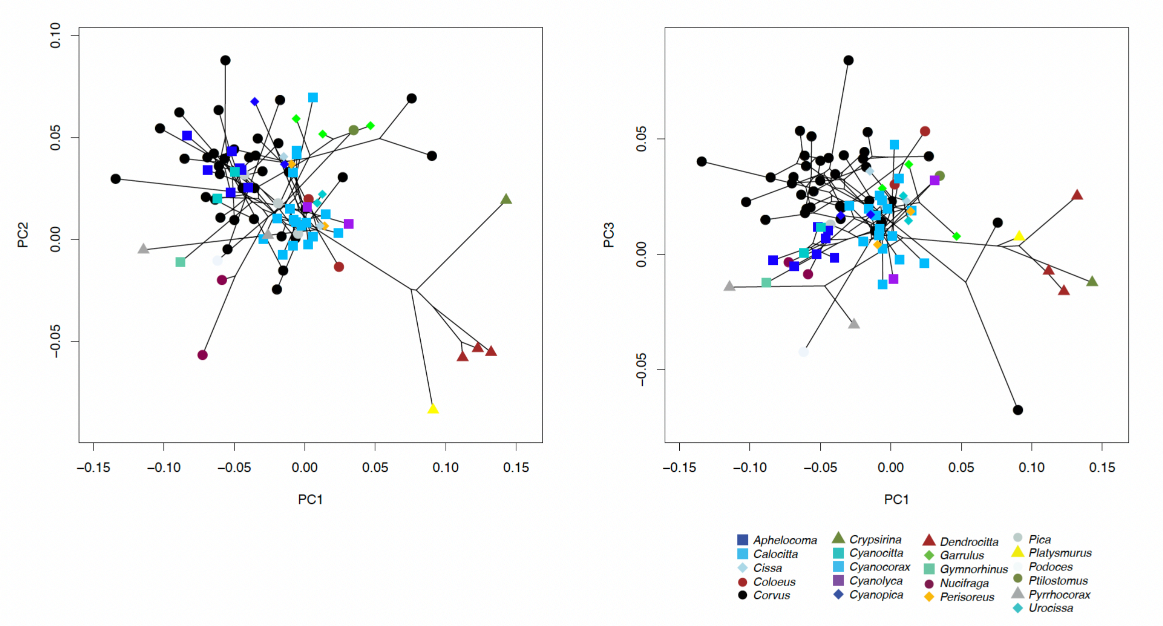

Beak shape morphospaces computed from allometry-free residuals (allometric free, geometric morphometric dataset - GMres). Given that beak shape variation was correlated to body size, we computed their allometry-free residuals to investigate if changes in rates of beak shape evolution were not driven by changes in rates of beak size evolution. Rates were estimated on the scores of a phylogenetic PCA derived from the allometric-free geometric morphometrics dataset (quantifying beak shape variation).

Supplementary Figure 13

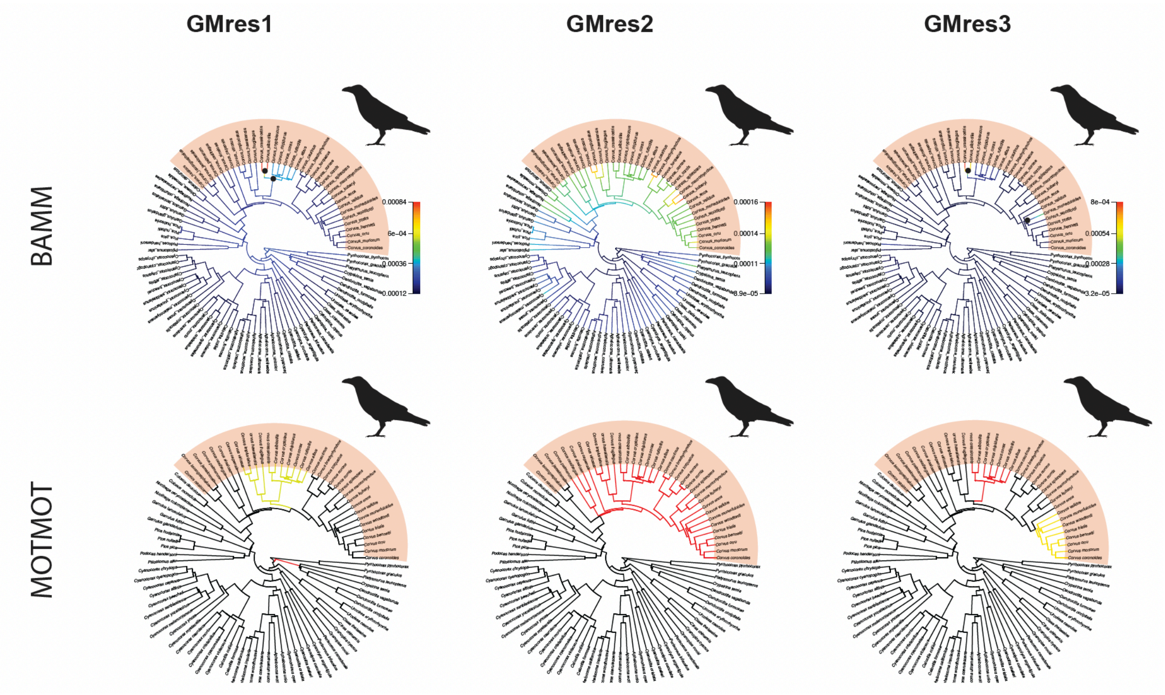

Rates of beak shape evolution (allometric free, geometric morphometric dataset - GMres dataset). Rates of beak shape evolution as calculated by BAMM are presented in the top row, with colours along branches denoting rates of phenotypic evolution. Dots indicate the locations of the most frequent shifts detected by BAMM (frequency > 0.2). Plots of rate heterogeneity as estimated by MOTMOT are presented in the bottom row. In all cases, the *Corvus* clade is highlighted in orange.

Supplementary Figure 14

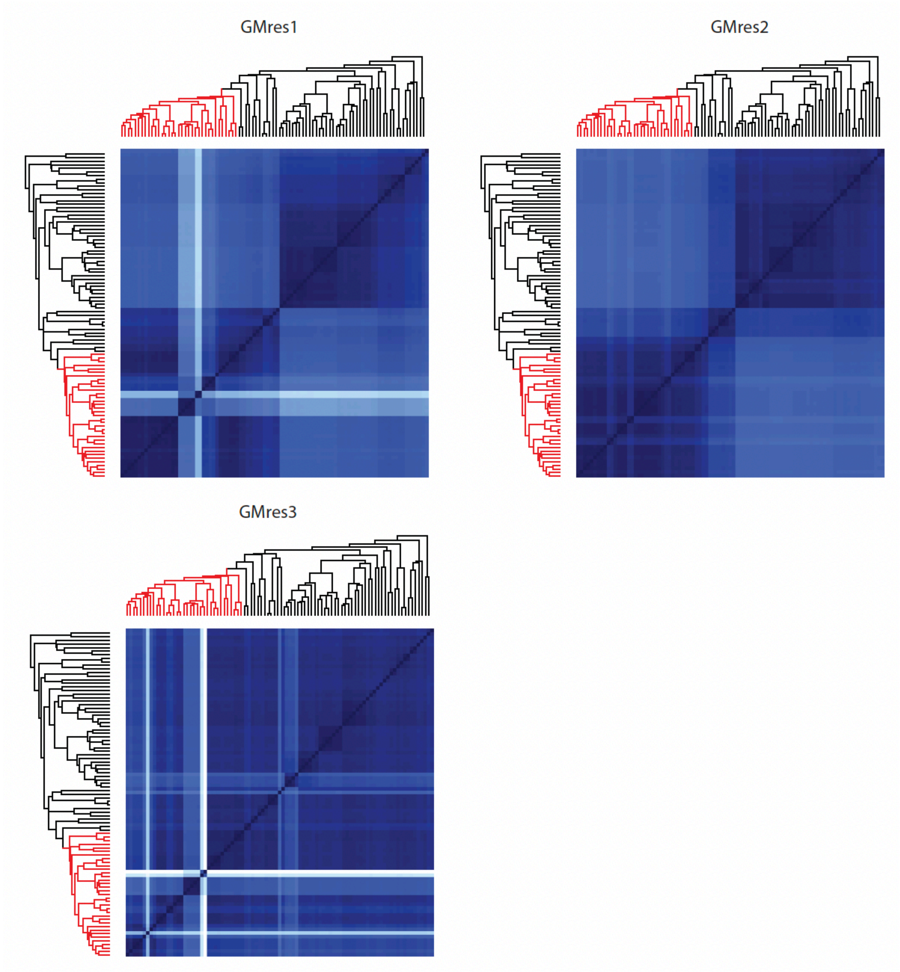

Cohort analyses for beak shape diversification in Corvidae (allometric free, geometric morphometric dataset, GMres dataset). The genus *Corvus* is identified in red within each phylogeny. The intensity of blue colouring in the macroevolutionary cohort matrices is proportional to the pairwise probability that two species share a common macroevolutionary rate regime (darker = higher probability).

Supplementary Figure 15

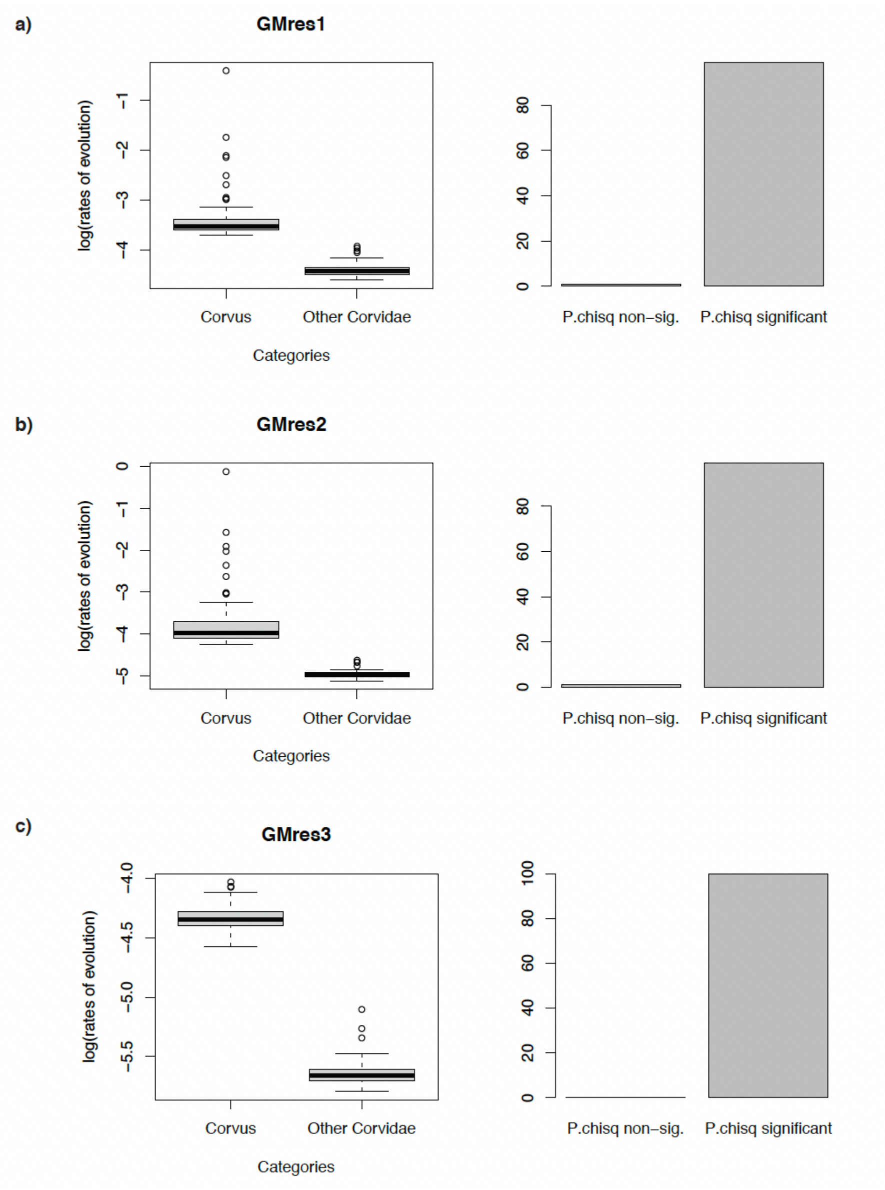

Rates of beak shape evolution (allometric free, geometric morphometric dataset - GMres dataset). The left panels show the rates of morphological evolution estimated by fitting, on each of the set posterior trees (100 trees), a Brownian rate variation ("noncensored") model (described in O'Meara et al. 2006; Evolution) that assumes two rates: a rate for the *Corvus* clade (including stem) and a rate for the remaining Corvidae. Box plots indicate median (middle line), 25th, 75th percentile (box), and 5th and 95th percentile (whiskers). The right panels show the proportion of significant p-values (one-tailed, based on 1,000 simulations) in likelihood ratio test against a χ^2 distribution (significant p-values imply rate variation).

Supplementary Figure 16

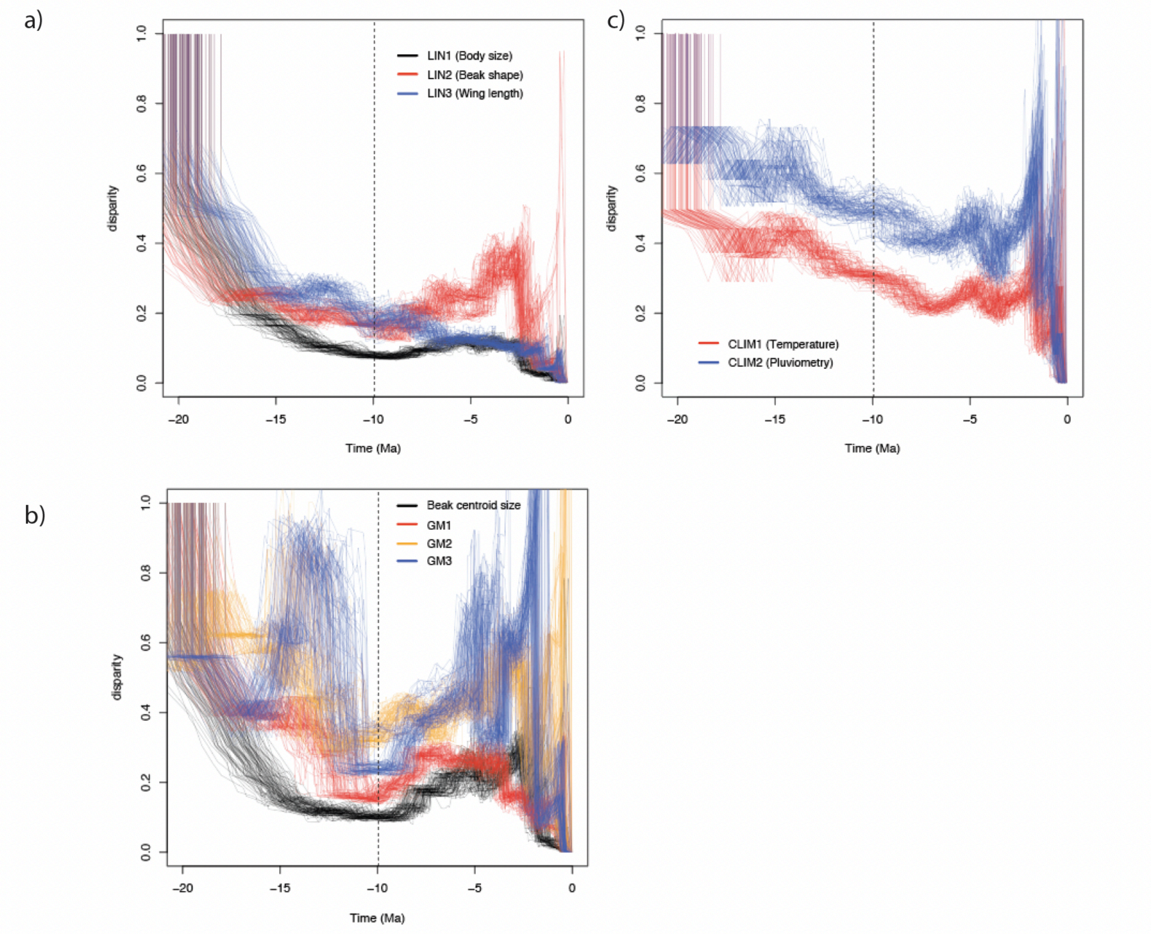

Disparity-though time analyses (DTT) across a sample of 100 trees obtained from the BEAST posterior distribution. (a) LIN refers to disparities derived from the dataset of linear measurements. (b) GM refer to disparities of beak shape evolution derived from the dataset obtained through geometric morphometrics. Beak centroid size is a proxy for beak size obtained from geometric morphometrics. (c) CLIM refers to climatic disparities.

Supplementary Figure 17

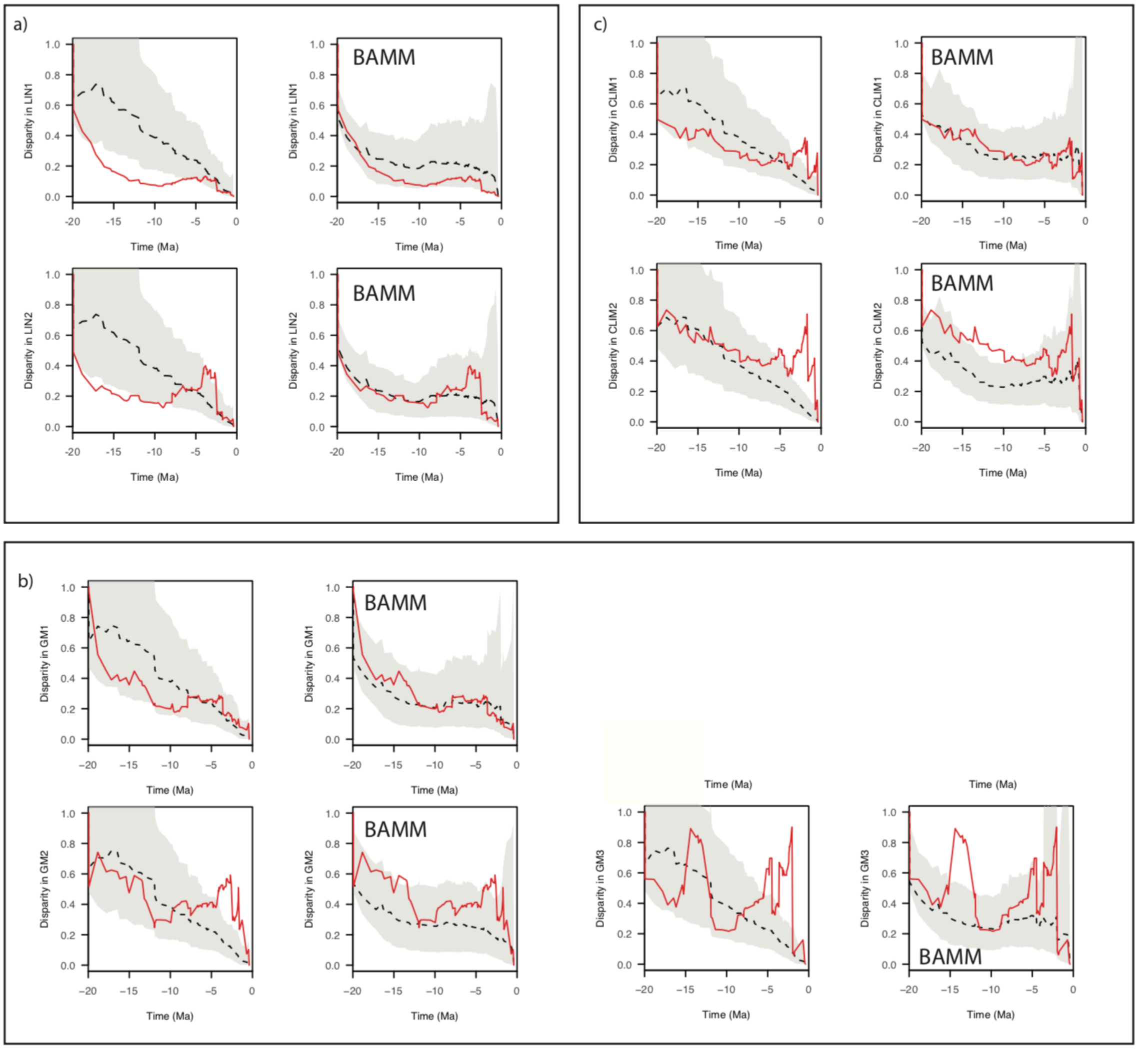

Simulated disparity-though time analyses (DTT) assuming rate heterogeneity. In all boxes, the left panels show the observed DTT pattern compared to the 95% CI (grey shading) and medians (discontinuous line) of DTT simulations assuming a single rate. Right panels (which include the label "BAMM") show the observed DTT pattern compared to the 95% CI (grey shading) and medians (discontinuous line) of DTT simulations with the rate heterogeneity estimated by BAMM (with rate acceleration within *Corvus*). (a) Linear measurements (LIN), (b) Beak shape through geometric morphometrics (GM) and (c) Climate (CLIM).

Supplementary Figure 18

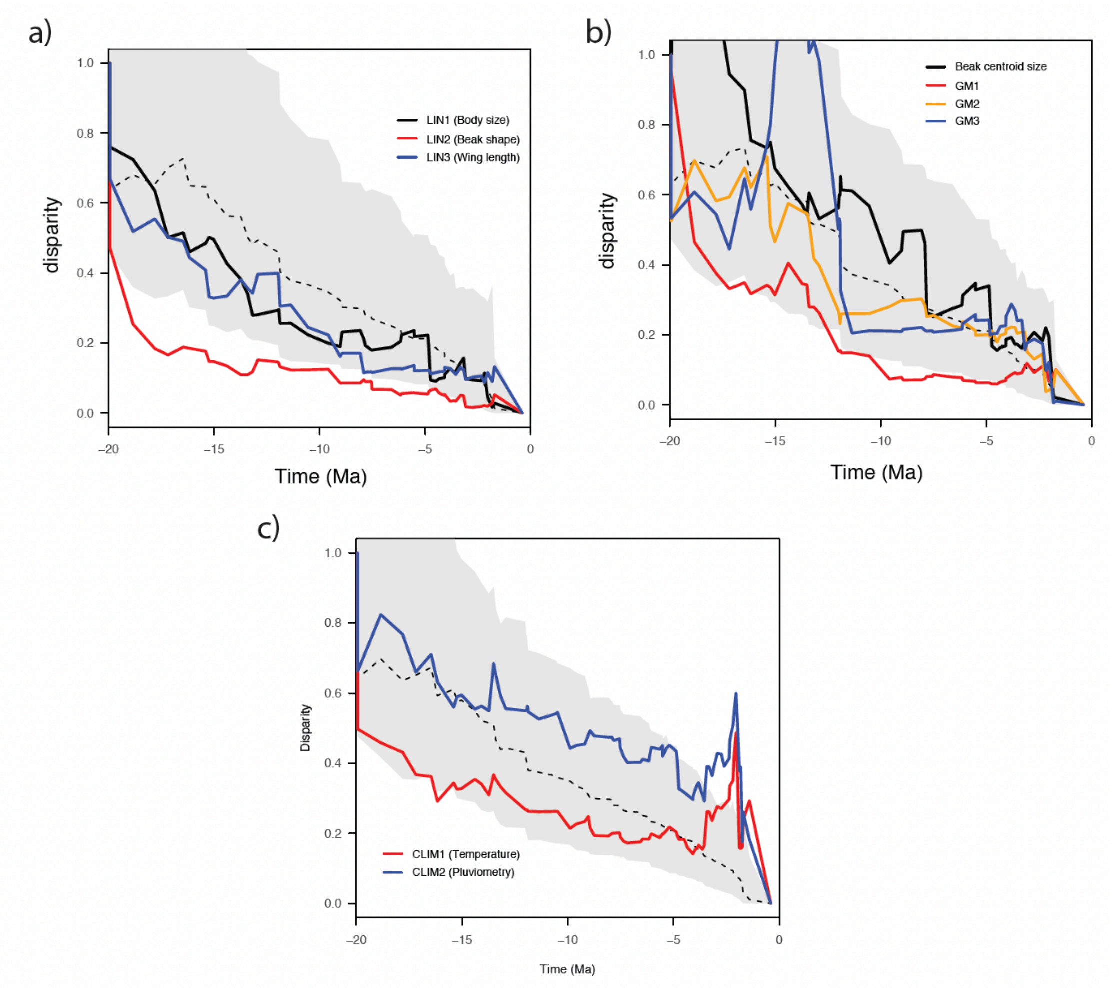

Disparity-though time analyses (DTT), excluding the genus *Corvus* from the phylogeny. Shaded regions depict the 95% confidence intervals from simulations under a null model with a single BM rate of evolution. LIN refers to disparities derived from the dataset of linear measurements. GM refer to disparities of beak shape evolution derived from the dataset obtained through geometric morphometrics. Beak centroid size is a proxy for beak size obtained from geometric morphometrics. CLIM refers to climatic disparities.

Supplementary Figure 19

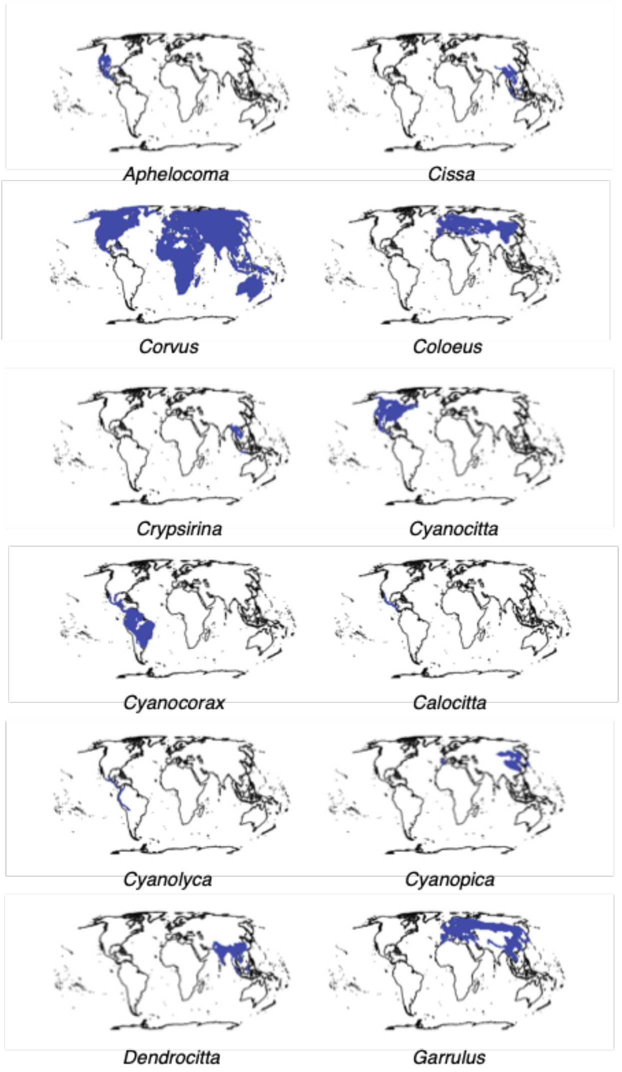

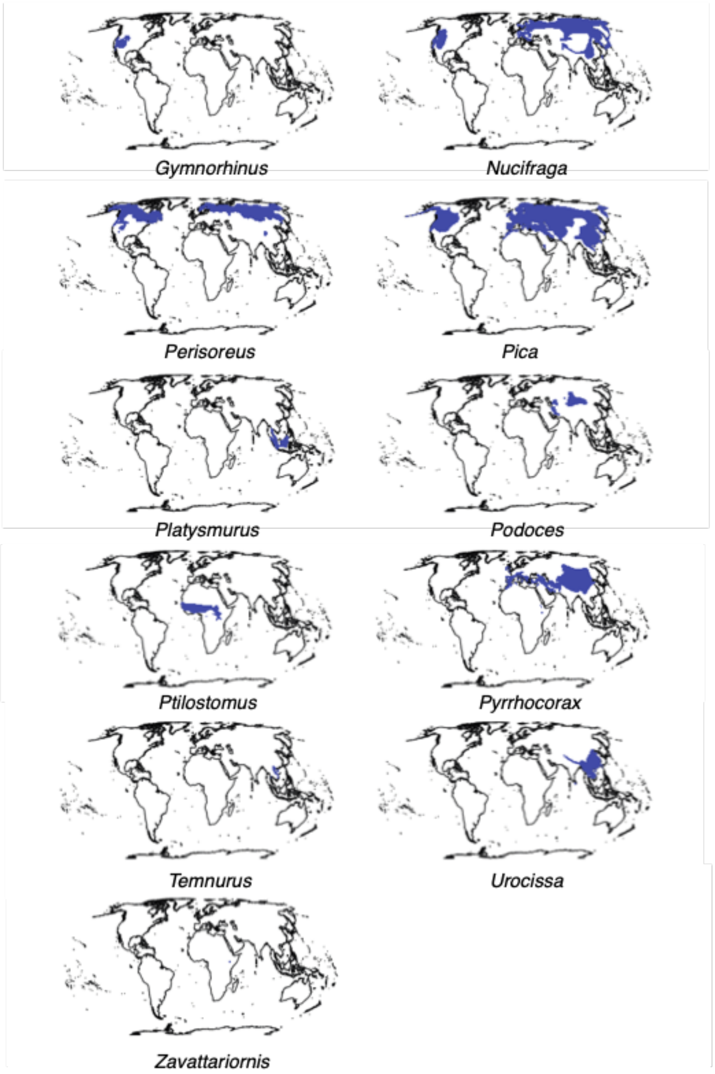

Distributions of all Corvidae genera. Maps are plotted using the Wagner IV equal-area projection. Data based on BirdLife International.

Supplementary Figure 20

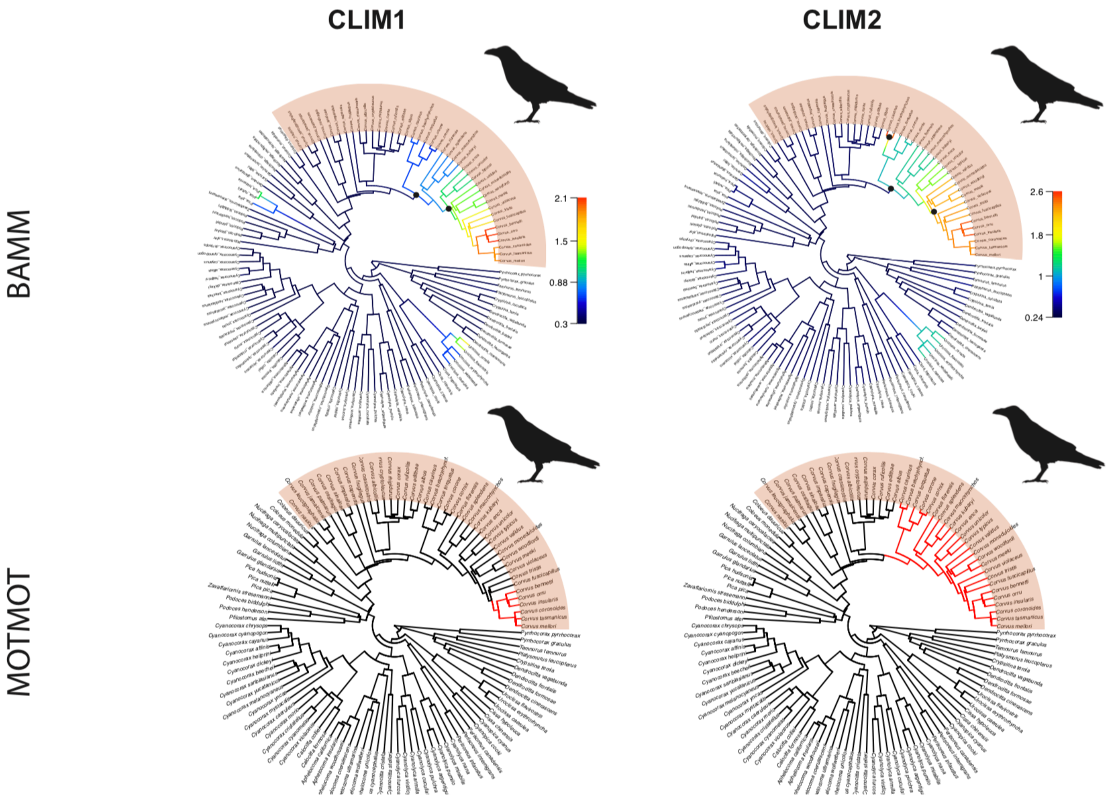

Rates of climate evolution (CLIM dataset). Rates of climate evolution as calculated by BAMM are presented in the top row, with colours along branches denoting rates of evolution. Dots depict the locations of the most frequent shifts detected by BAMM (frequency > 0.05). Plots of rate heterogeneity as estimated by MOTMOT are presented in the bottom row. In all cases, the *Corvus* clade is highlighted in orange.

Supplementary Figure 21

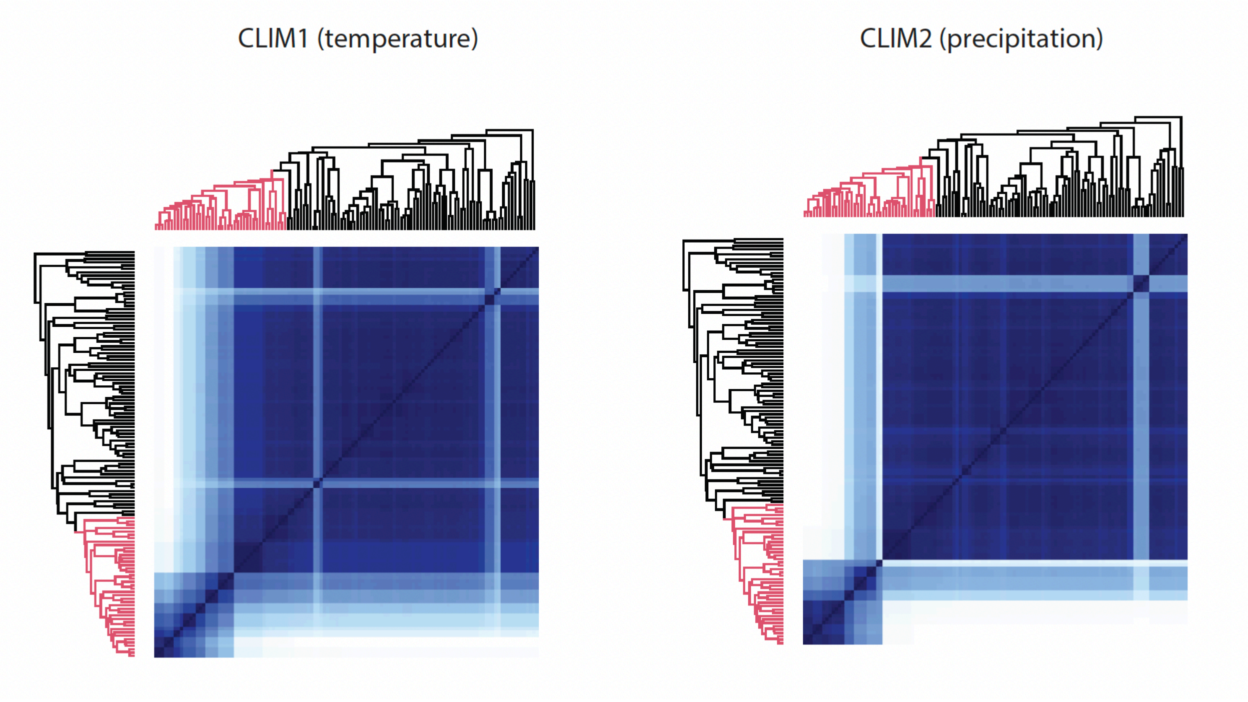

Cohort analyses for climate diversification in Corvidae (CLIM dataset). The genus *Corvus* is identified in red within each phylogeny. The intensity of blue colouring in the macroevolutionary cohort matrices is proportional to the pairwise probability that two species share a common macroevolutionary rate regime (darker = higher probability).

Supplementary Figure 22.

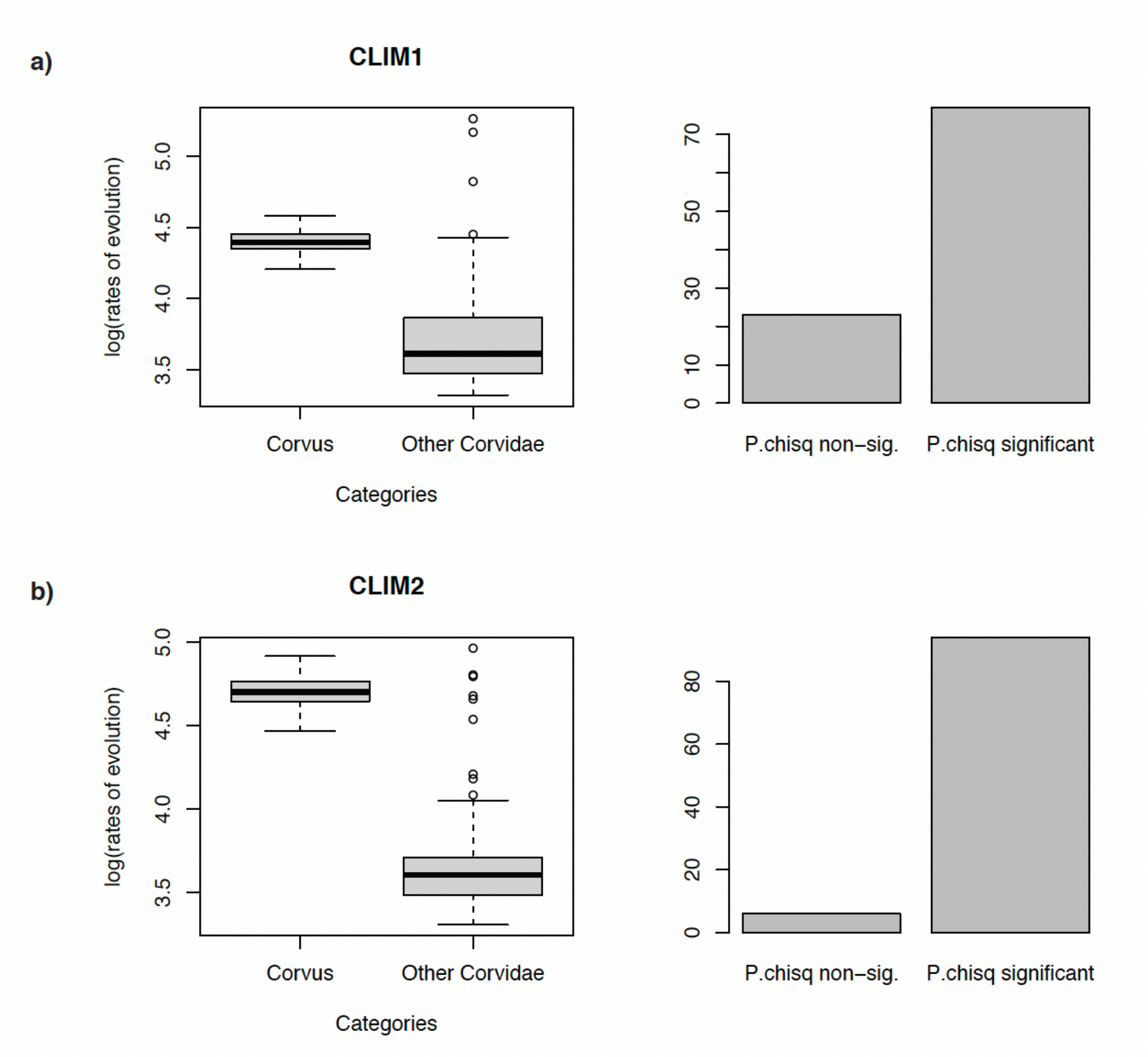

Rates of climate evolution (CLIM dataset). The left panels show the rates of climatic evolution estimated by fitting, on each of the set posterior trees (100 trees), a Brownian rate variation ("noncensored") model (described in O'Meara et al. 2006; Evolution) that assumes two rates: a rate for the *Corvus* clade (including stem) and a rate for the remaining Corvidae. Box plots indicate median (middle line), 25th, 75th percentile (box), and 5th and 95th percentile (whiskers). The right panels show the proportion of significant p-values (one-tailed, based on 1,000 simulations) in likelihood ratio test against a χ^2 distribution (significant p-values imply rate variation).

Supplementary Figure 23

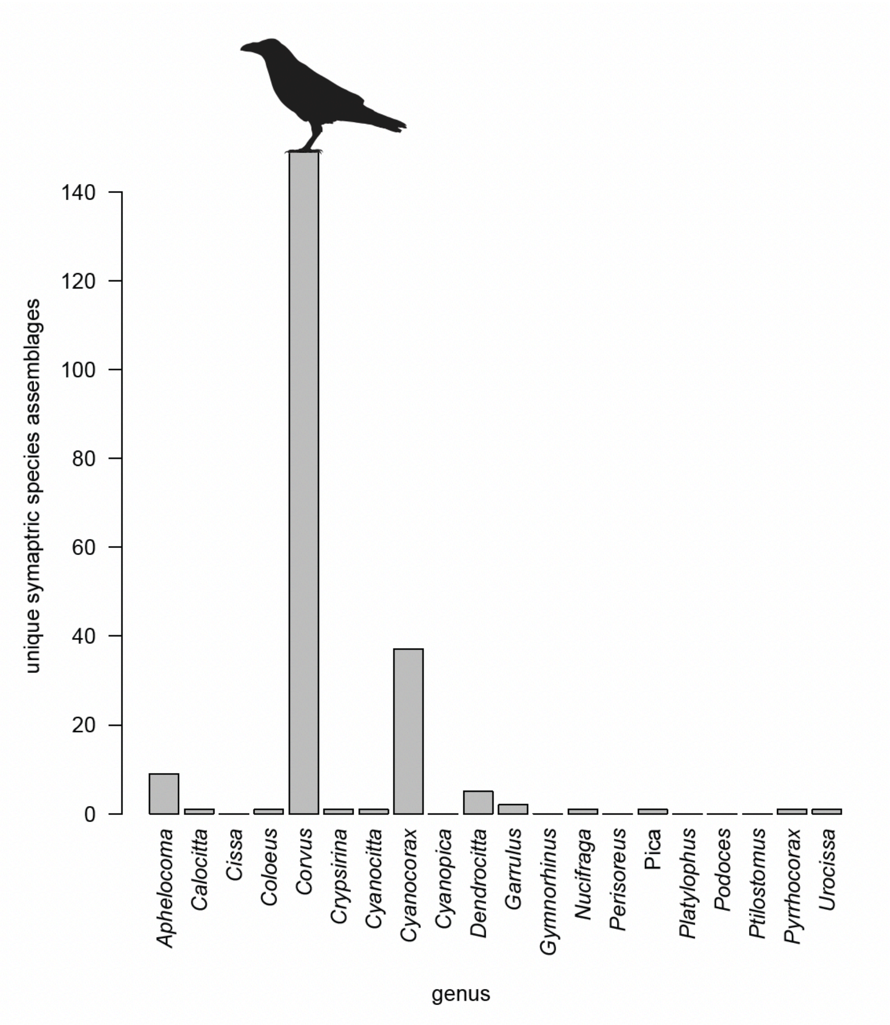

Number of unique sympatric assemblages among genera in the family Corvidae. Based on eBird data at a resolution of 11 x 11 km.

Supplementary Figure 24

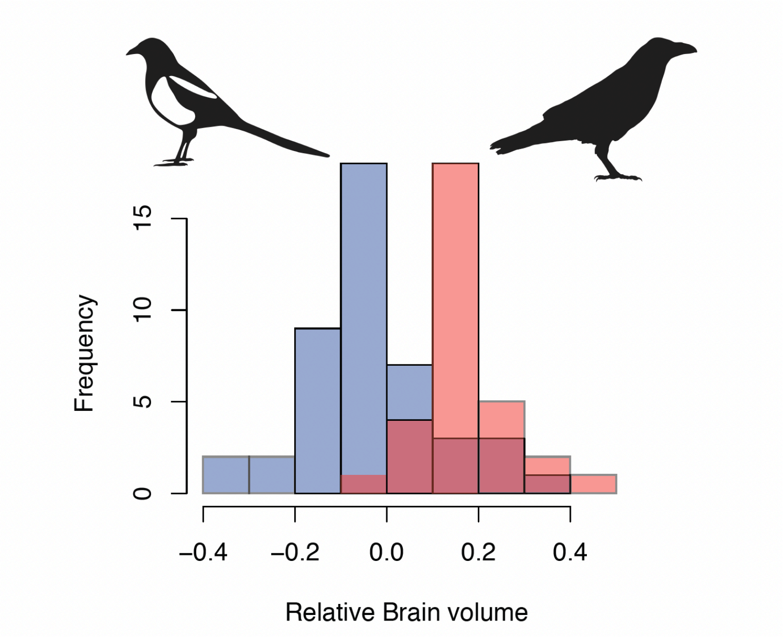

Relative brain size variation in the family Corvidae. *Corvus* histogram is coloured in red, all other Corvidae, in blue.

Supplementary Figure 25

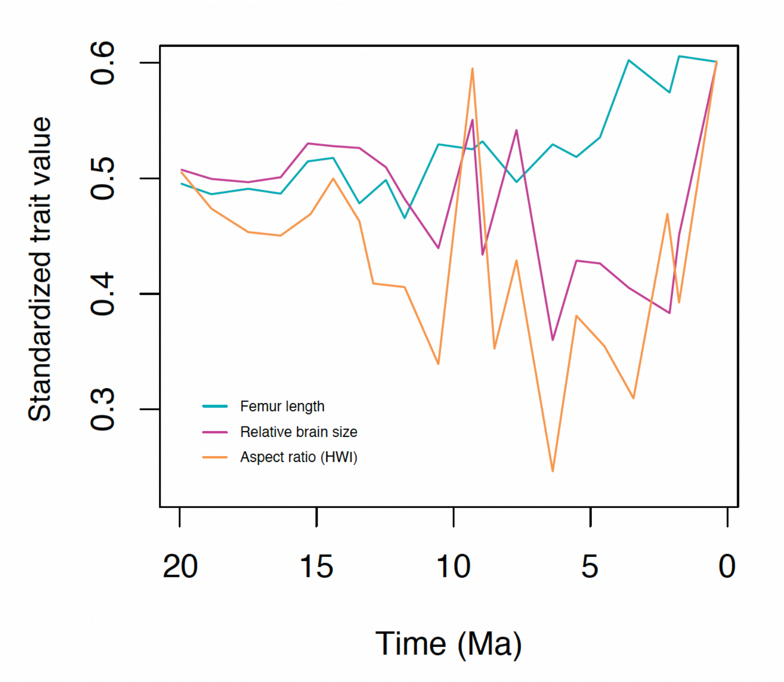

Variation of body size (femur length), "hand-wing index" (HWI), and relative brain size, through time excluding *Corvus*. Variation through time of the mean ancestral body size (as estimated from femur length), HWI, and relative brain size, when the genus *Corvus* is excluded from the phylogeny.

Supplementary Figure 26

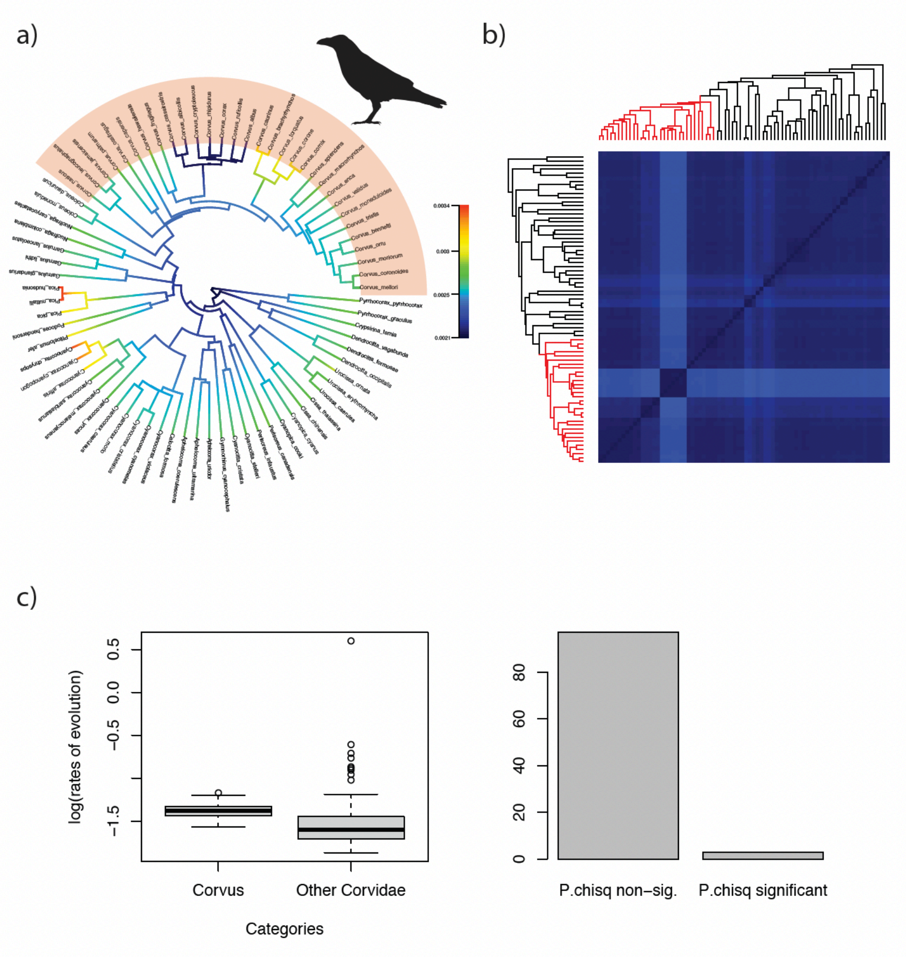

Rates of diversification of relative brain size in Corvidae. (a) Rates of evolution as calculated by BAMM, with colours along branches denoting rates of phenotypic evolution. (b) Cohort analyses for relative brain size in the family Corvidae. The genus *Corvus* is highlighted in red within each phylogeny. The intensity of blue colouring in the macroevolutionary cohort matrices is proportional to the pairwise probability that two species share a common macroevolutionary rate regime (darker = higher probability). (c) The left panel show the rates of relative brain size evolution estimated by fitting, on each of the set posterior trees (100 trees), a Brownian rate variation ("noncensored") model (described in O'Meara et al. 2006; Evolution) that assumes two rates: a rate for the *Corvus* clade (including stem) and a rate for the remaining Corvidae. Box plots indicate median (middle line), 25th, 75th percentile (box), and 5th and 95th percentile (whiskers). The right panels show the proportion of significant p-values (one-tailed, based on 1,000 simulations) in likelihood ratio test against a χ^2 distribution (significant p-values imply rate variation).

Supplementary Figure 27

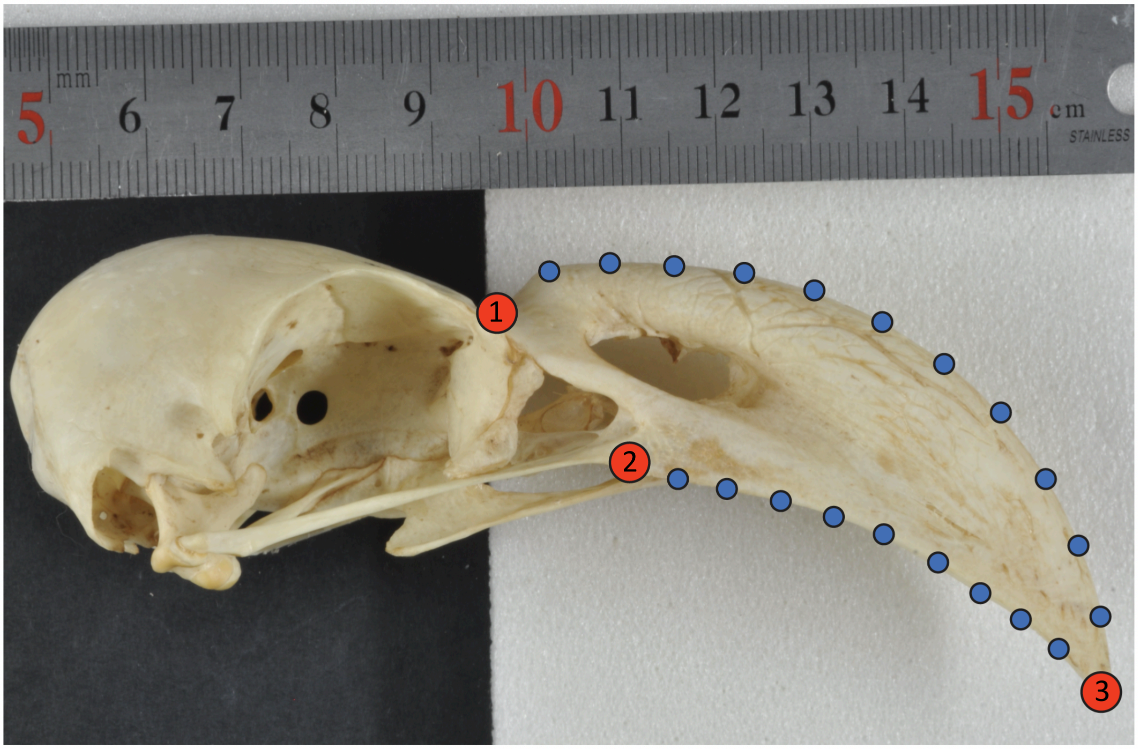

Landmark placement to obtain the geometric morphometric dataset on beak shape. Locations for the beak landmarks and semi-landmarks are respectively depicted in red and blue.

Supplementary Figure 28

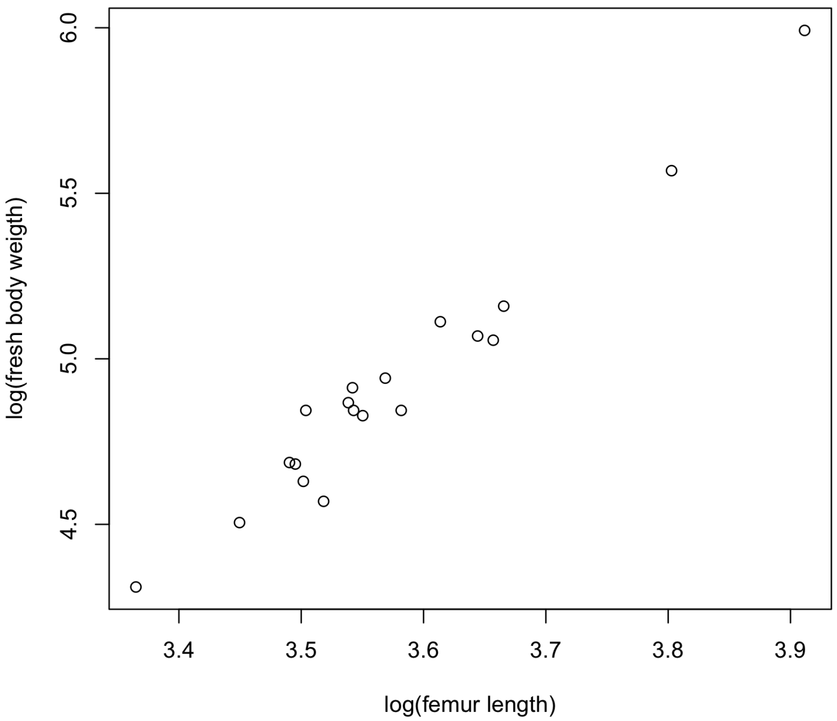

Covariance between fresh weight and femur length in Corvidae. Scatterplot showing the strong covariation between the femur length and the fresh weight of the same specimen (n=19) across multiple species in the family Corvidae (see raw data in Supplementary Data File 1).

Supplementary Figure 29

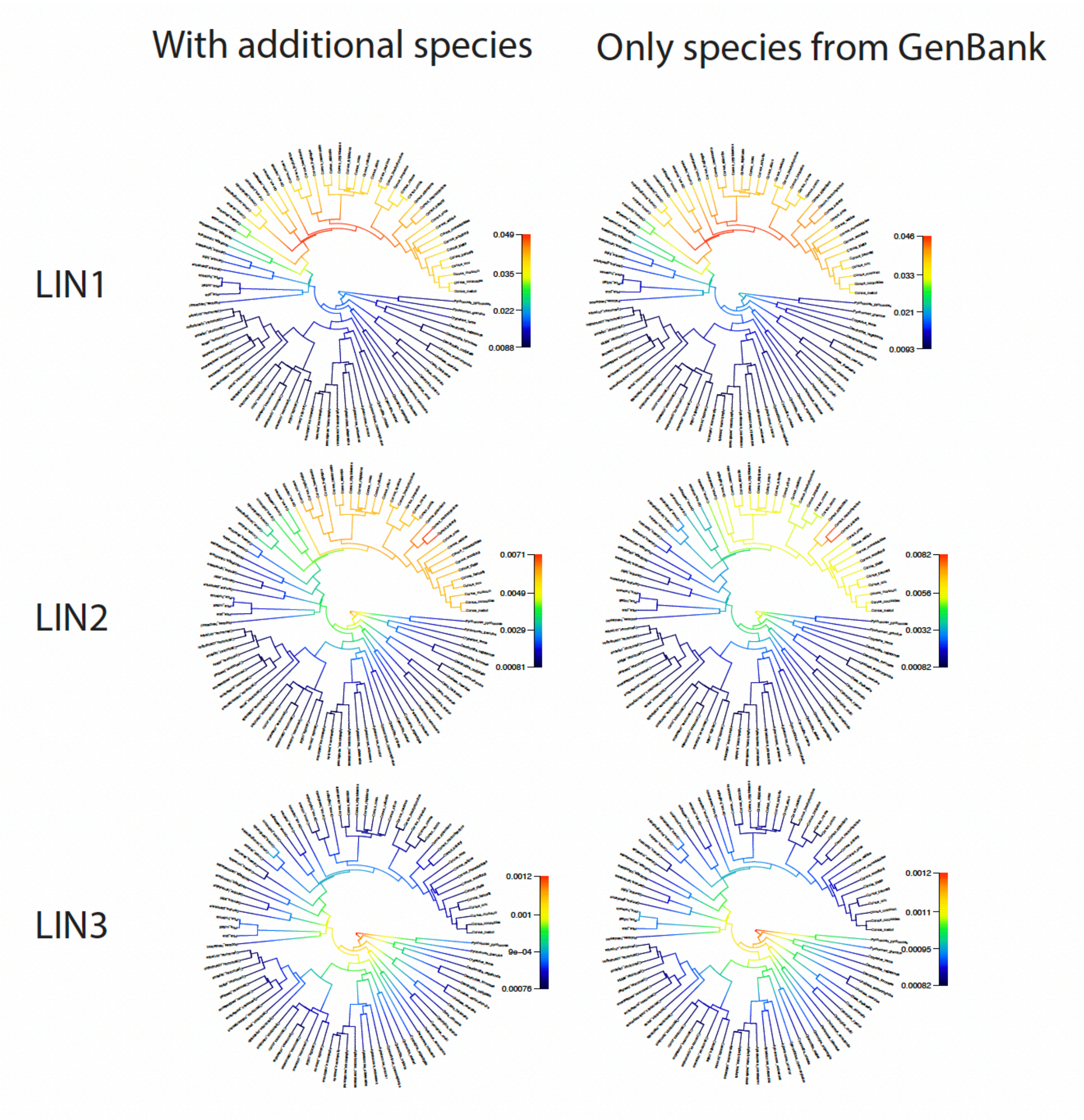

Analyses of the robustness of our findings to the inclusion of species with no molecular data (linear measurements, LIN). Mean rates of evolution as detected by BAMM are presented for analyses that included species that were manually included in the phylogeny using taxonomic and biogeographic criteria (left) and for analyses that only included species available in GenBank (right).

Supplementary Figure 30

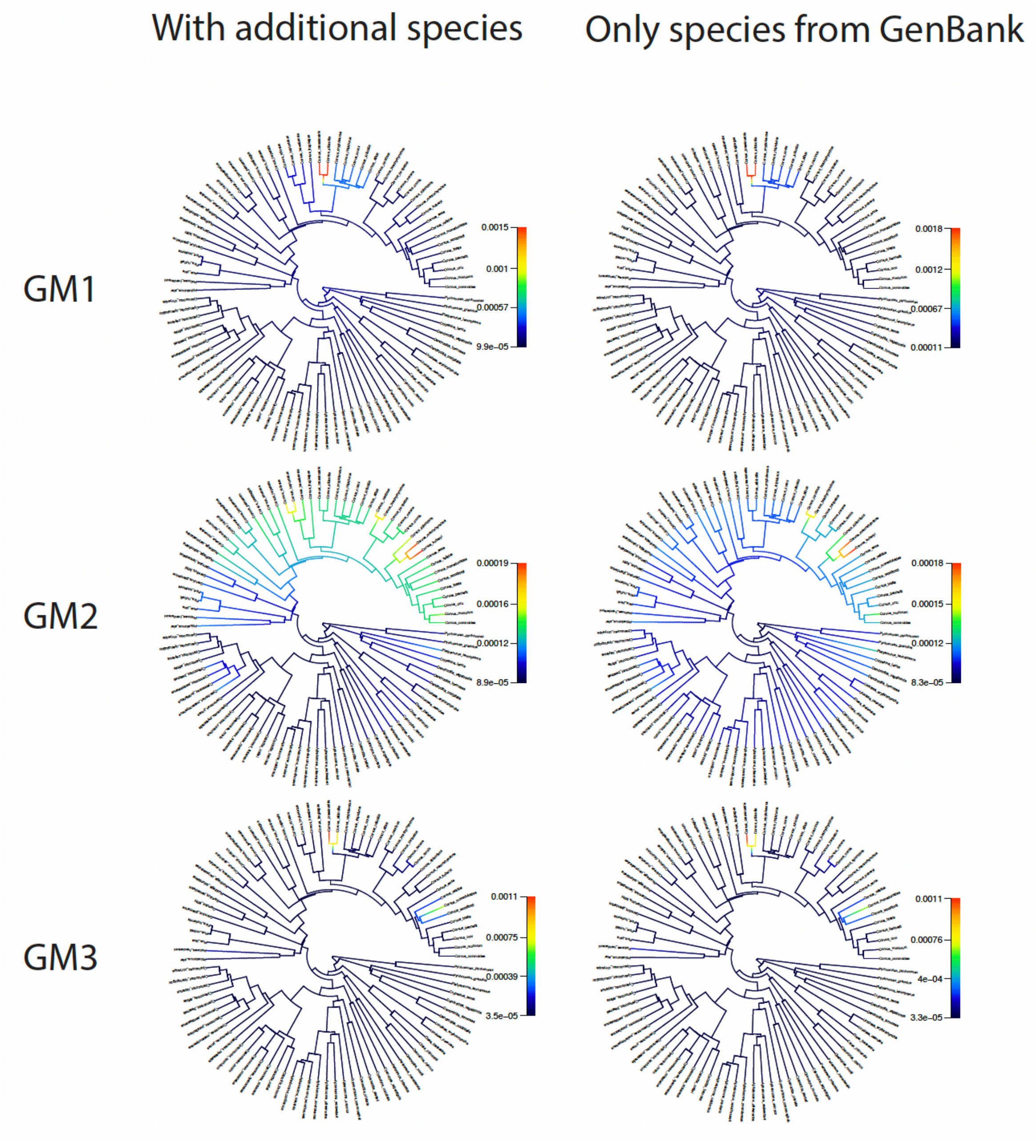

Analyses of the robustness of our findings to the inclusion of species with no molecular data (geometric morphometrics, GM). Mean rates of evolution as detected by BAMM are presented for analyses that included species that were manually included in the phylogeny using taxonomic and biogeographic criteria (left) and for analyses that only included species available in GenBank (right).

Supplementary Figure 31

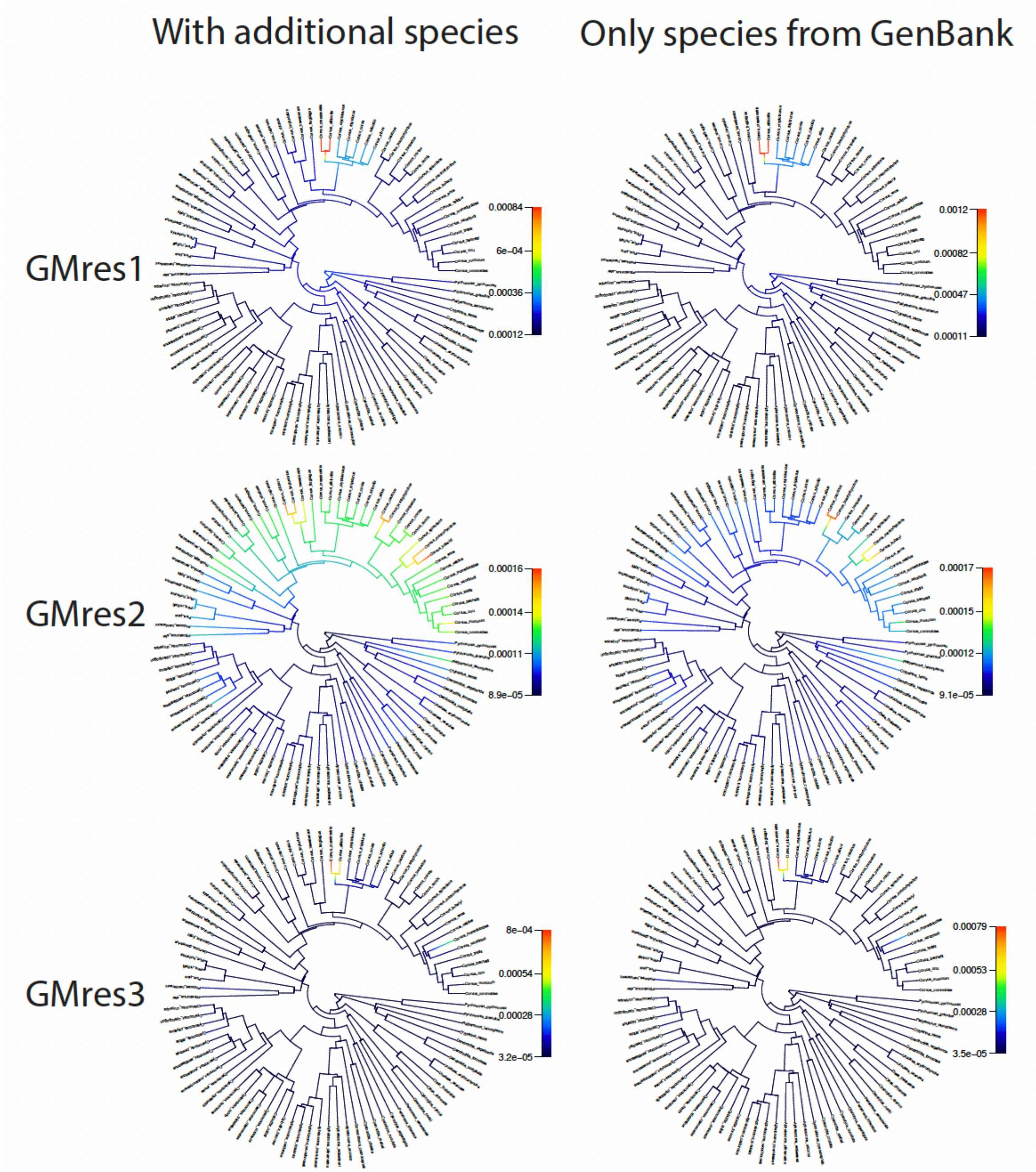

Analyses of the robustness of our findings to the inclusion of species with no molecular data (geometric morphometrics – residuals, GMres). Mean rates of evolution as detected by BAMM are presented for analyses that included species that were manually included in the phylogeny using taxonomic and biogeographic criteria (left) and for analyses that only included species available in GenBank (right).

Supplementary Figure 32

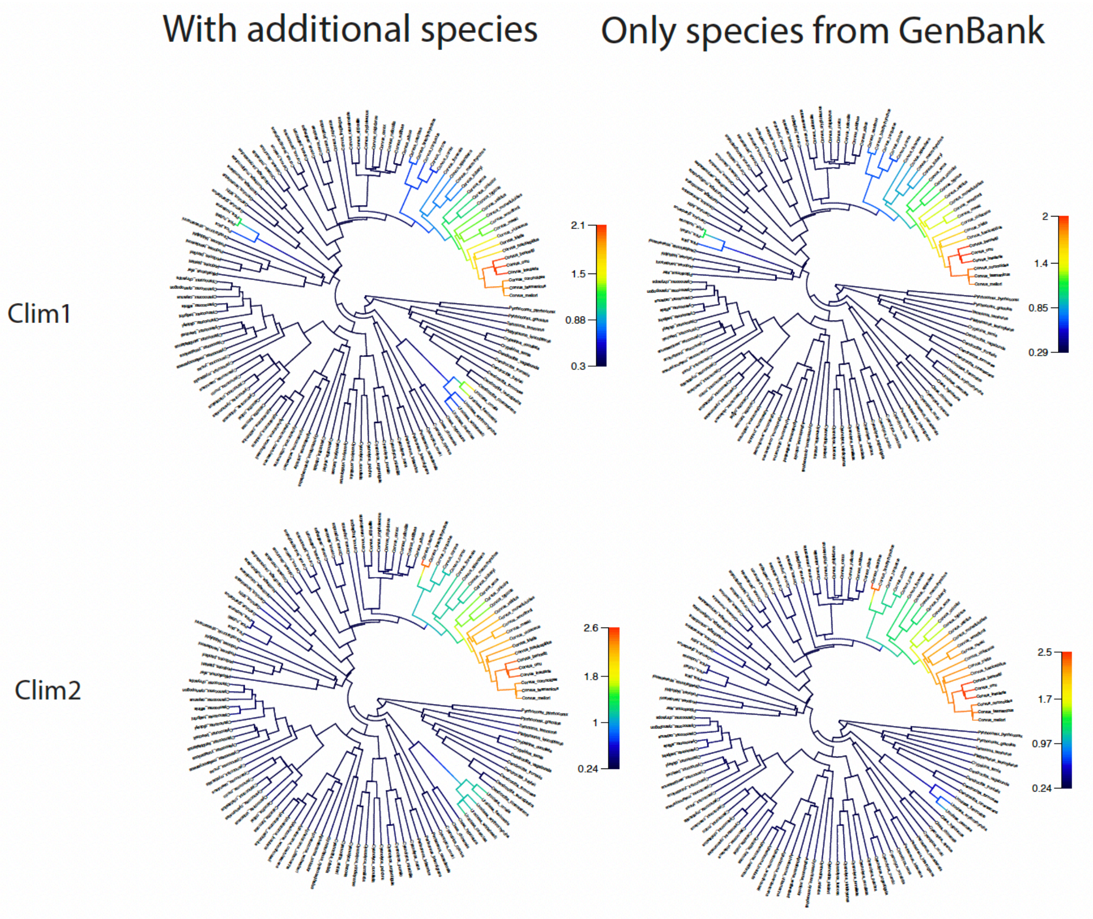

Analyses of the robustness of our findings to the inclusion of species with no molecular data (climate). Mean rates of evolution as detected by BAMM are presented for analyses that included species that were manually included in the phylogeny using taxonomic and biogeographic criteria (left) and for analyses that only included species available in GenBank (right).

Supplementary tables

Supplementary Table 1

dataset	model	GIC
LIN1	BM	-1849.253
LIN2	EB	-1848.134
LIN3	OU	-1847.249
GM1	BM	-51321.49
GM2	EB	-51500.31
GM3	OU	-50926.57
GM.res1	BM	-50734.87
GM.res2	EB	-50915.94
GM.res3	OU	-50390.67

Relative model support (GIC values) for phylogenetic PCAs on the different datasets. LIN = Linear dataset, GM = beak shape geometric morphometrics dataset, GMres = beak shape geometric morphometrics dataset (allometric-free).

Supplementary Table 2

Variable	PC1	PC2	PC3
Beak length	0.35	-0.19	0.08
Beak heigth	0.32	-0.66	-0.20
Beak width	0.34	-0.43	-0.13
Tarsus length	0.34	0.42	-0.59
Tibiotarsus length	0.37	0.34	-0.19
Femur length	0.37	0.12	-0.08
Humerus length	0.37	0.13	0.45
Ulna length	0.36	0.16	0.59

Loadings of the phylogenetic PCA for the linear dataset (LIN).

Supplementary Table 3

Variable name	Variable description	PC1	PC2
bio 1	Annual Mean Temperature	-1183.42	-587.75
bio 2	Mean Diurnal Range (Mean of monthly (max temp - min temp))	-207.80	-1004.02
bio 3	Isothermality (BIO2/BIO7) (×100)	-1137.50	-42.21
bio 4	Temperature Seasonality (standard deviation ×100)	1075.70	-29.72
bio 5	Max Temperature of Warmest Month	-947.85	-790.38
bio 6	Min Temperature of Coldest Month	-1258.82	-376.96
bio 7	Temperature Annual Range (BIO5-BIO6)	993.59	-284.36
bio 8	Mean Temperature of Wettest Quarter	-983.02	-599.02
bio 9	Mean Temperature of Driest Quarter	-1141.09	-490.06
bio 10	Mean Temperature of Warmest Quarter	-1009.00	-719.31
bio 11	Mean Temperature of Coldest Quarter	-1244.58	-450.42
bio 12	Annual Precipitation	-1020.41	799.77
bio 13	Precipitation of Wettest Month	-978.83	556.37
bio 14	Precipitation of Driest Month	-640.15	893.13
bio 15	Precipitation Seasonality (Coefficient of Variation)	200.53	-424.77
bio 16	Precipitation of Wettest Quarter	-1000.63	583.99
bio 17	Precipitation of Driest Quarter	-675.30	909.30
bio 18	Precipitation of Warmest Quarter	-799.65	655.90
bio 19	Precipitation of Coldest Quarter	-740.29	750.13

Loadings of the PCA for climate variables (CLIM).

SI REFERENCES

1. James, H. F. & Olson, S. L. Descriptions of thirty-two new species of birds from the Hawaiian Islands: Part II. Passeriformes. *Ornithol. Monogr.* 1–88 (1991).

2. Bonacum, J., O'Grady, P. M., Kambysellis, M. & DeSalle, R. Phylogeny and age of diversification of the *planitibia* species group of the Hawaiian *Drosophila*. *Mol. Phylogenet. Evol.* **37**, 73–82 (2005).
