# Supplementary material for: Niche expansion and adaptive divergence in the global radiation of crows and ravens
Source: Nat Commun. 2022 Apr 21;13:2086. doi: 10.1038/s41467-022-29707-5 (PMC9023458; doi:10.1038/s41467-022-29707-5)
Supplement: Supplementary file 2 — Description of Additional Supplementary Files [file 41467_2022_29707_MOESM2_ESM.pdf]

File Name: Supplementary Data 1
Description: Species, genes, and GenBank accession numbers of the sequences appended to the DNA matrix generated in Jønsson et al. 2016. Appended sequences highlighted in orange.

File Name: Supplementary Data 2
Description: Linear measurements (beak, upper and lower limbs) obtained in this study (in cm). AMNH = American Museum of Natural History (New York), BMNH = British Museum of Natural History (Tring), FMNH = Field Museum of Natural History (Chicago), MNHN = Muséum National d'Histoire Naturelle (Paris), MVZ = Museum of Vertebrate Zoology (Berkeley) and SI = Smithsonian Institution (Washington DC). Fresh weights (in grams) were obtained from specimen's labels.

File Name: Supplementary Data 3
Description: TPS file containing the mean landmark coordinates used to describe beak shape for each species.

File Name: Supplementary Data 4
Description: Average endocranial volumes (ml) collected and used in this study.

File Name: Supplementary Software
Description: Custom code produced in this study.
