# Supplementary material for: Niche expansion and adaptive divergence in the global radiation of crows and ravens
Source: Nat Commun. 2022 Apr 21;13:2086. doi: 10.1038/s41467-022-29707-5 (PMC9023458; doi:10.1038/s41467-022-29707-5)
Supplement: Supplementary file 8 — Reporting Summary [file 41467_2022_29707_MOESM8_ESM.pdf]

nature research

Corresponding author(s): Joan Garcia-Porta

Last updated by author(s): Feb 12, 2022

Reporting Summary

Nature Research wishes to improve the reproducibility of the work that we publish. This form provides structure for consistency and transparency in reporting. For further information on Nature Research policies, see our Editorial Policies and the Editorial Policy Checklist.

Statistics

For all statistical analyses, confirm that the following items are present in the figure legend, table legend, main text, or Methods section.

n/a	Confirmed	
☒	☐	The exact sample size (*n*) for each experimental group/condition, given as a discrete number and unit of measurement
☐	☒	A statement on whether measurements were taken from distinct samples or whether the same sample was measured repeatedly
☐	☒	The statistical test(s) used AND whether they are one- or two-sided<br>*Only common tests should be described solely by name; describe more complex techniques in the Methods section.*
☐	☒	A description of all covariates tested
☐	☒	A description of any assumptions or corrections, such as tests of normality and adjustment for multiple comparisons
☐	☒	A full description of the statistical parameters including central tendency (e.g. means) or other basic estimates (e.g. regression coefficient) AND variation (e.g. standard deviation) or associated estimates of uncertainty (e.g. confidence intervals)
☐	☒	For null hypothesis testing, the test statistic (e.g. *F*, *t*, *r*) with confidence intervals, effect sizes, degrees of freedom and *P* value noted<br>*Give P values as exact values whenever suitable.*
☐	☒	For Bayesian analysis, information on the choice of priors and Markov chain Monte Carlo settings
☒	☐	For hierarchical and complex designs, identification of the appropriate level for tests and full reporting of outcomes
☒	☐	Estimates of effect sizes (e.g. Cohen's *d*, Pearson's *r*), indicating how they were calculated

Our web collection on statistics for biologists contains articles on many of the points above.

Software and code

Policy information about availability of computer code

Data collection	We collected linear measurements of the appendicular skeleton using the software ImageJ v1.52.
Data analysis	Phylogenetic analysis were conducted in BEAST v.2.4.8, TranslatorX (no version provided by authors, http://translatorx.co.uk) and tracer v. 1.6. Statistical analyses were conduced in BAMM v2.5.0 and in R with the packages: BAMMtools v2.1.7, geiger v2.0.6.4, Castor v1.6.7, geomorph v3.2.1, mvMORPH v1.1.1, alphahull v2.1, phytools 0.7-70, motmot v2.1.3, raster v3.4-10, ape v5.5 and phylolm v2.6.2, as well as custom code, provided in Supplementary Software.

For manuscripts utilizing custom algorithms or software that are central to the research but not yet described in published literature, software must be made available to editors and reviewers. We strongly encourage code deposition in a community repository (e.g. GitHub). See the Nature Research guidelines for submitting code & software for further information.

Data

Policy information about availability of data

All manuscripts must include a data availability statement. This statement should provide the following information, where applicable:

- Accession codes, unique identifiers, or web links for publicly available datasets
- A list of figures that have associated raw data
- A description of any restrictions on data availability

Morphological data is available as Supplementary Data Files 1-4. Climatic data is available in the WorldClim database (http://worldclim.org). Shapefiles of the distribution of each species of corvid were downloaded from BirdLife International (http://datazone.birdlife.org/species/requestdis). The coordinates of observations of corvid species were obtained from eBird (https://ebird.org/science/use-ebird-data/download-ebird-data-products).

Field-specific reporting

Please select the one below that is the best fit for your research. If you are not sure, read the appropriate sections before making your selection.

- [ ] Life sciences
- [ ] Behavioural & social sciences
- [x] Ecological, evolutionary & environmental sciences

For a reference copy of the document with all sections, see nature.com/documents/nr-reporting-summary-flat.pdf

Ecological, evolutionary & environmental sciences study design

All studies must disclose on these points even when the disclosure is negative.

Study description	We studied the global diversification of the genus Corvus, comparing phenotypic, climatic and species diversification rates between the genus Corvus and the rest of Corvidae.
Research sample	Regarding morphology, we measured almost all species available in the osteological collections of the British Museum of Natural History, Museum National of Histoire Naturelle, Smithsonian Institution, Field Museum of Natural History, the Museum of Vertebrate Zoology and the American Museum of Natural History. Distribution data were obtained from all species available in BirdLife. Regarding molecular phylogenetics we appended the genetic dataset of the superfamily Corvoidea compiled by Jonsson et al (2016).
Sampling strategy	Regarding morphology, when possible we tried to include at least 2 specimens per species (male and female when possible). We sampled linear measurements for 93 species (237 specimens), beak shape for 96 species (213 specimens) and endocraneal volume for 76 species (197 specimens) of corvids. Distribution data consisted in 121 species. Regarding genetic data, we obtained 354 new sequences from GenBank, not included in Jonsson et al (2016).
Data collection	Linear and geometric morphometric data was collected from pictures taken at osteological collections using the software ImageJ. Pictures and measurement on pictures was collected by JGP. We measured brain volumes by filling the brain cavity of skulls of museum specimens with 1 mm glass microballoons (GB 01, conservation resources UK limited) of known density and weighing these microballoons with a digital scale at a 0.01 grams of precision. We converted weights to volumes using their known density. Brain cavities were measured by JGP and FS.
Timing and spatial scale	September 2017. Collection of genetic data from GenBank. Data collection in the British Museum of Natural History (London): 16/01/2017 – 31/01/2017. Data collection in the Museum National d'Histoire Naturelle (Paris): 12/03/2017 – 29/03/2017. Data collection in the Field Museum of Natural History (Chicago): 01/05/2017 - 15/05/2017 and 04/02/2019 - 08/02/2019. Data collection in the Smithsonian Institution (Washington DC): 17/05/2017 - 22/05/2017 and 11/02/2019 - 26/02/2019. Data collection in the American Museum of Natural History (New York): 28/02/2019 - 15/03/2019. Data collection in the Museum of Vertebrate Zoology (Berkeley): 21/04/2019 - 04/05/2019.
Data exclusions	We solely excluded specimens that did not have all osteological elements measured (so they could not be included in the analyses).
Reproducibility	We repeated measurements 3 times, making sure that measurements taken on pictures were consistent. Statistical analysis were performed using different approaches, all of them with consistent results.
Randomization	We report here the results of a variety of comparative analysis on morphological and behavioral data. Because no actual experiments were performed, randomization of test subjects does not apply
Blinding	Measurements on pictures were obtained without knowing which species was being measured (to avoid biases at obtaining data). The two main analysis used (such as BAMM and MOTMOT) estimate diversification patterns blindly without prior knowledge or information on the shifts on diversification rates.

Did the study involve field work? [ ] Yes [x] No

Reporting for specific materials, systems and methods

We require information from authors about some types of materials, experimental systems and methods used in many studies. Here, indicate whether each material, system or method listed is relevant to your study. If you are not sure if a list item applies to your research, read the appropriate section before selecting a response.

Materials & experimental systems

n/a	Involved in the study	
☒	☐	Antibodies
☒	☐	Eukaryotic cell lines
☒	☐	Palaeontology and archaeology
☐	☒	Animals and other organisms
☒	☐	Human research participants
☒	☐	Clinical data
☒	☐	Dual use research of concern

Methods

n/a	Involved in the study	
☒	☐	ChIP-seq
☒	☐	Flow cytometry
☒	☐	MRI-based neuroimaging

Animals and other organisms

Policy information about studies involving animals; ARRIVE guidelines recommended for reporting animal research

Laboratory animals	No Laboratory animals were used in this study.
Wild animals	No wild animals were used in this study.
Field-collected samples	No field-collected samples were used in this study.
Ethics oversight	As we worked on dead, preserved, specimens from public museum collections (collected many years before this study), no animal suffering was involved in our research, therefore, no ethical guidance or approval is necessary.

Note that full information on the approval of the study protocol must also be provided in the manuscript.
